# Human‐modified canids in human‐modified landscapes: The evolutionary consequences of hybridization for grey wolves and free‐ranging domestic dogs

**DOI:** 10.1111/eva.13257

**Published:** 2021-06-21

**Authors:** Małgorzata Pilot, Andre E. Moura, Innokentiy M. Okhlopkov, Nikolay V. Mamaev, Ninna H. Manaseryan, Vahram Hayrapetyan, Natia Kopaliani, Elena Tsingarska, Abdulaziz N. Alagaili, Osama B. Mohammed, Elaine A. Ostrander, Wiesław Bogdanowicz

**Affiliations:** ^1^ Museum and Institute of Zoology Polish Academy of Sciences Warsaw Poland; ^2^ Institute of Biological Problems of Cryolithozone Siberian Branch of Russian Academy of Sciences Yakutsk Russia; ^3^ Scientific Center of Zoology and Hydroecology National Academy of Sciences Yerevan Armenia; ^4^ Green Artsakh Biosphere Complex SNCO Artsakh Armenia; ^5^ Institute of Ecology Ilia State University Tbilisi Georgia; ^6^ BALKANI Wildlife Society Sofia Bulgaria; ^7^ KSU Mammals Research Chair Department of Zoology King Saud University Riyadh Saudi Arabia; ^8^ Cancer Genetics and Comparative Genomics Branch National Human Genome Research Institute National Institutes of Health Bethesda MD USA

**Keywords:** adaptive introgression, domestic dog, domestication, grey wolf, human‐induced evolution, introgressive hybridization

## Abstract

Introgressive hybridization between domestic animals and their wild relatives is an indirect form of human‐induced evolution, altering gene pools and phenotypic traits of wild and domestic populations. Although this process is well documented in many taxa, its evolutionary consequences are poorly understood. In this study, we assess introgression patterns in admixed populations of Eurasian wolves and free‐ranging domestic dogs (FRDs), identifying chromosomal regions with significantly overrepresented hybrid ancestry and assessing whether genes located within these regions show signatures of selection. Although the dog admixture proportion in West Eurasian wolves (2.7%) was greater than the wolf admixture proportion in FRDs (0.75%), the number and average length of chromosomal blocks showing significant overrepresentation of hybrid ancestry were smaller in wolves than FRDs. In wolves, 6% of genes located within these blocks showed signatures of positive selection compared to 23% in FRDs. We found that introgression from wolves may provide a considerable adaptive advantage to FRDs, counterbalancing some of the negative effects of domestication, which can include reduced genetic diversity and excessive tameness. In wolves, introgression from FRDs is mostly driven by drift, with a small number of positively selected genes associated with brain function and behaviour. The predominance of drift may be the consequence of small effective size of wolf populations, which reduces efficiency of selection for weakly advantageous or against weakly disadvantageous introgressed variants. Small wolf population sizes result largely from human‐induced habitat loss and hunting, thus linking introgression rates to anthropogenic processes. Our results imply that maintenance of large population sizes should be an important element of wolf management strategies aimed at reducing introgression rates of dog‐derived variants.

## 1 INTRODUCTION

Domestication is a striking example of human‐induced evolution. Adaptation to new ecological niches created by humans as well as artificial selection pressures have resulted in extensive changes in domesticated organisms, including morphology, physiology, reproduction and trophic position (Milla et al., 2015; Solberg et al., 2020; Wilkins et al., 2014). The effects of these changes are not limited to domesticated organisms, but extend into the entire biosphere (Larson & Fuller, 2014). In particular, domesticated organisms have a profound effect on their wild relatives through resource competition, pathogen transfer and hybridization (Lescureux & Linnell, 2014; Turcotte et al., 2017). This effect is becoming increasingly pronounced with the ongoing population growth of domestic animals and plants (Foley et al., 2005; Gompper, 2014; Thornton, 2010).

Introgressive hybridization between domestic animals and their wild relatives has an important effect on gene pools and phenotypic traits of both groups. This process can be considered as a form of human‐induced evolution, as it results from a combination of human‐induced processes that have taken place on different timescales, from ancient domestication processes to recent ecosystem changes affecting distribution, density and patterns of interbreeding between sympatric or parapatric taxa (Crispo et al., 2011; Grabenstein & Taylor, 2018). Although the process of hybridization between wild and domestic taxa is extensively documented (reviewed in McFarlane & Pemberton, 2019), its evolutionary consequences are poorly understood. In general, introgressive hybridization can have negative consequences such as the loss of unique adaptations or extinction via genetic swamping (Todesco et al., 2016). Alternatively, there may be positive consequences such as transmission of adaptive variation between species (e.g., Jones et al., 2018; Oziolor, 2019; Song et al., 2011), genetic rescue (Whiteley et al., 2015) and adaptive evolution (when hybridization‐derived traits facilitate adaptation to novel environmental conditions; Hedrick, 2013).

Introgression from domesticated taxa is thought to have predominantly negative consequences for wild populations, given that genetic and phenotypic variation resulting from domestication may be deleterious for wild animals, and numerical preponderance of domestic animals over their wild relatives facilitates genetic swamping. In some instances, however, variants of immune system genes originating from domestic animals were shown to have positive fitness effects in their wild relatives. In North American grey wolves, a dog‐derived variant of beta‐defensin 103 immune system gene has increased in frequency and geographic range since its introduction through hybridization more than 1500 years ago, and currently shows signature of balancing selection in several populations (Anderson et al., 2009; Schweizer et al., 2018). Fitness measures such as lifespan and lifetime reproductive success show strong selective advantage for heterozygous individuals carrying the dog‐derived variant (Coulson et al., 2011), which is likely related to an enhanced immune response (Schweizer et al., 2018). The Alpine ibex (*Capra ibex ibex*) has only two MHC *DRB* exon 2 alleles, one of which was shown to originate from domestic goats (*Capra aegagrus hircus*) (Grossen et al., 2014). The resulting improved immune response could have contributed to the successful reintroduction of this species following its near extinction (Grossen et al., 2014). Although these examples show that genetic variation transferred from domestic animals may be adaptive in their wild relatives in some circumstances, it is unknown how anthropogenic introgression affects genome‐wide adaptive variation in wild populations. The evolutionary consequences of introgression from wild populations to their domesticated relatives are also largely unknown.

Cross‐breeding populations of the grey wolf (*Canis lupus*) and the domestic dog (*Canis lupus familiaris*) provide an excellent model system for studying the consequences of anthropogenic introgression. As the first domesticated species (Larson & Fuller, 2014), the dog has been living in human‐modified habitats longer than any other domestic animal and acquired multiple adaptations to living as a human commensal. A notable example is the increased copy number of the alpha‐2B‐amylase (*AMY2B*) gene in comparison with wolves, which has facilitated digestion of starch‐rich food (Axelsson et al., 2013) following the spread of prehistoric agriculture (Arendt et al., 2016). The size of the dog population is positively correlated with the size of the human population and currently reaches one billion individuals worldwide, of which about 75% are free‐ranging (Gompper, 2014). In continental parts of the European Union, the number of dogs in rural areas was estimated at 18.4 million (Gompper, 2014), while the number of wolves in the same region at the same time was estimated at 12,000 (Chapron et al., 2014), resulting in a dog to wolf ratio of approximately 1500:1. This implies that most wolves are likely to encounter a dog during their lifetime. Such encounters can result in dogs being killed by wolves, or in some instances in mating and production of hybrid offspring (Lescureux & Linnell, 2014).

Wolf‐dog hybrids are fertile and can reproduce with both wolves and dogs, resulting in introgression within both wolf and dog populations (e.g., Hindrikson et al., 2017; Kopaliani et al., 2014; Pilot et al., 2019). During the expansion of domestic dogs throughout the world from their original domestication region(s), hybridization with local wolf populations may have facilitated dog adaptation to local environments. For instance, dogs native to the Tibetan Plateau carry a variant of the *EPAS1* gene associated with adaptation to the low‐oxygen environment, which derives from an ancient adaptive introgression from Tibetan grey wolves (Miao et al., 2017). The notion that wolf admixture can have beneficial effects for dogs resulted in intentional cross‐breeding, which was practised by human societies across the world (Lescureux, 2018).

In contrast, introgression from domestic dogs into grey wolves is considered a conservation threat, as the accumulation of dog‐derived gene variants in wolves' gene pools can lead to a gradual loss of genetic distinctiveness and unique adaptive variation (Donfrancesco et al., 2019; Hindrikson et al., 2017). A general expectation exists that heritable traits originating from domestic dogs are maladaptive for wild wolves living in their natural ecosystems, since dogs have been subject to artificial selection and are adapted to the ecological niche of human commensal. However, for wolves living in human‐dominated landscapes, some traits originating from domestic dogs may become advantageous. Currently, little is known about the effects of introgression of dog‐derived variants into the gene pool of wolf populations, with the exception of adaptive introgression of a dog‐derived *CBD103* variant in North American wolves described above (Anderson et al., 2009; Schweizer et al., 2018).

The most likely sources of such introgression are free‐ranging dogs (FRDs) inhabiting rural areas. Eurasian FRDs are not a product of admixture between breeds, but constitute a distinct and older genetic group (Pilot et al., 2015; Shannon et al., 2015). Although FRDs depend on anthropogenic food, they can survive without having any continued direct interaction with humans, they have no constraints in mate choice and therefore are not subject to ongoing artificial selection. Accordingly, their genomes display lower levels of deleterious genetic variation than pure‐bred dogs (Marsden et al., 2016). FRDs and pure‐bred dogs show signatures of diversifying selection in genes related to reproduction, immunity and chemosensory perception, which may reflect adaptations of FRDs to independent survival (Pilot et al., 2016). Some adaptive traits of FRDs may become beneficial to wild canids living in human‐dominated landscapes, if acquired via hybridization. For example, wolves living in habitats heavily transformed by humans are likely to be increasingly exposed to encounters with domestic dogs and dog‐derived immune system gene variants could enhance immunity of wolves to dog‐derived infectious diseases.

The aim of this study is to (a) assess introgression patterns in Eurasian populations of wolves and FRDs, (b) identify chromosomal regions with a significant deficiency or excess of introgressed ancestry in both canids and (c) assess whether genes placed within these regions are under selection, and are associated with phenotypic traits which differ between wolves and FRDs and for which introgression may result in increased fitness.

## 2 MATERIALS AND METHODS

### 2.1 Dataset

This study focused on Eurasian grey wolves and FRDs, known from earlier studies to have undergone introgressive hybridization (e.g., Fan et al., 2016; Hindrikson et al., 2017; Pilot et al., 2015). The dataset analysed in this study was obtained by merging genome‐wide SNP genotypes from worldwide populations of grey wolves and FRDs as well as pure‐bred domestic dogs from publicly available datasets (Cronin et al., 2015; Fitak et al., 2018; Frantz et al., 2016; Pilot et al., 2015, 2019; Stronen et al., 2015; Vaysse et al., 2011; Vernau et al., 2013). Although this study was focused on wolf‐dog hybridization in Eurasia, we included data from North American grey wolves and pure‐bred dogs in the analysed dataset to ensure sufficient representation of nonadmixed individuals. North American wolves show very limited genome‐wide introgression from dogs (Fitak et al., 2018; Pilot et al., 2018) and most dog breeds of European origin show very limited or no wolf admixture (Pilot et al., 2015, 2019; vonHoldt et al., 2010).

All datasets included in the analyses were generated using the CanineHD Whole‐Genome Genotyping BeadChip (Illumina). The population structure analysis for the merged dataset using admixture (Alexander et al., 2009) showed that individuals originating from different datasets but representing the same dog breeds or wolf populations cluster together, demonstrating that the merging was performed correctly. The CanineHD BeadChip produces genotypes at 167,989 autosomal SNP loci and 5660 X chromosome SNP loci. Some of the published datasets reported a reduced set of loci compared with the total number included in the CanineHD BeadChip, and/or did not report X chromosome loci. Therefore, the final merged and pruned dataset included data for 106,549 autosomal SNP loci. This dataset included 1526 individuals (506 wolves and 1020 dogs), each with a genotyping rate above 90% (Table S1).

**FIGURE 1:**
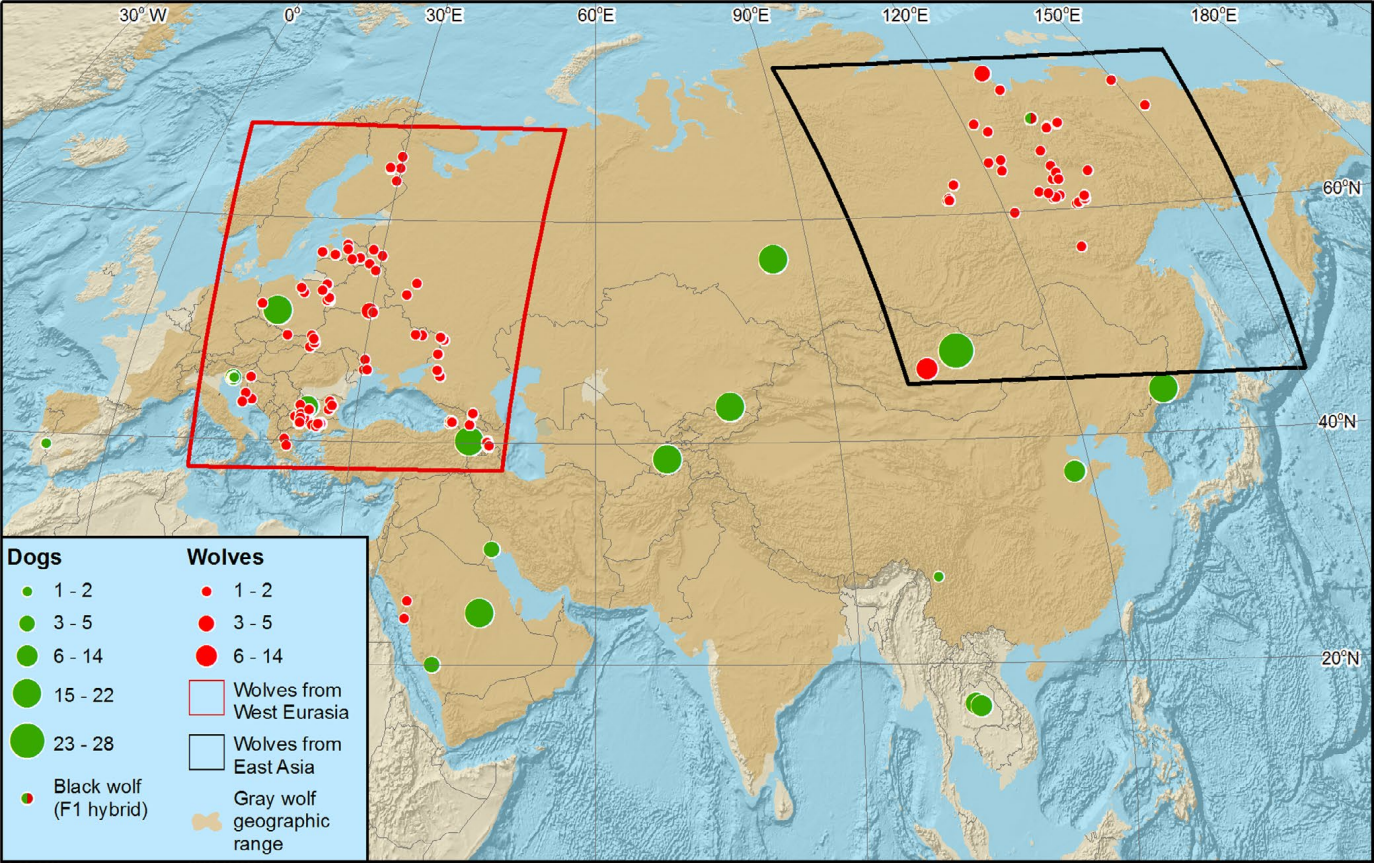
Distribution of samples of Eurasian grey wolves (red circles) and free‐ranging dogs (green circles) analysed in this study. Geographic locations of samples are precise except Mongolian wolves and free‐ranging dogs from China and Portugal, which have approximate locations. The number of samples collected from the same locations is reflected by the circle size. The introgression pattern analysis was carried out for West Eurasian wolves (marked with the red frame) and all free‐ranging dogs shown on the map that carried introgressed chromosomal fragments. Among East Asian wolves (black frame), only four admixed individuals were found, including an F1 hybrid. Grey wolf geographic range drawn according to Boitani et al. (2018) and Wang, Ma, et al. (2016)

With a few exceptions, sampling for this dataset does not cover exactly the same regions for wolves and FRDs (Figure 1). However, Eurasian free‐ranging domestic dogs originate from a recent geographic expansion, dated at about 15,000 years ago (Wang, Zhai, et al., 2016) and do not show strong population structure across Eurasia (Pilot et al., 2015). Modern grey wolves were also shown to originate from an expansion of a single source population that began approximately 25,000 years ago (Loog et al., 2020) and display a limited population structure across Eurasia (e.g., Ersmark et al., 2016; Pilot, Dąbrowski, et al., 2014; Pilot et al., 2019), with the exception of Indian and Himalayan wolves (Sharma et al., 2004), that are not included in this research. Given that we have a broad geographic coverage for both wolves and dogs, our dataset provides a strong representation of genetic variability of both canids across Eurasia. Therefore, the inference of admixture between wolves and dogs should not be affected by the lack of exact geographic overlap between their sampling sites.

The Illumina Canine HD BeadChip was developed in collaboration with the LUPA Consortium and was based on a dataset that, in addition to dog breeds, included 15 grey wolves from Europe and North America (Vaysse et al., 2011). The wolves showed 118,256 segregating sites, of which 1471 sites were variable in wolves but not in any dog breed analysed. Because of the small number of wolves included in the reference panel, loci that show variation in wolves but not dogs are underrepresented in the chip, which may affect the admixture inference. However, the Canine HD BeadChip was previously used in studies on dog introgression into wolves (e.g., Galaverni et al., 2017) and wolf introgression into dogs (Caniglia et al., 2018). This last study was focused on Czechoslovakian wolfdogs, and the inferred admixture proportions were consistent with the breed history. Moreover, the inference of admixture proportions in both wolf and dog populations based on the Canine HD BeadChip (Pilot et al., 2019) is consistent with the inference from whole‐genome data (Fan et al., 2016; Freedman et al., 2014). Nevertheless, we note that the underrepresentation of genetic variation specific to wolves may result in the reduced power to detect wolf introgression into dogs compared with the reverse.

### 2.2 Ancestry block analysis

plink software (www.cog‐genomics.org/plink/1.9; Chang et al., 2015) was used to remove from the dataset SNPs with low variability (MAF < 0.01) and those with >10% missing data. For the purpose of the analysis in lamp (see below), we also removed SNPs in strong linkage disequilibrium (LD; *r* ^2^ > 0.1). We assessed the presence of chromosomal ancestry blocks derived from hybridization in individual canids in two steps, initially using the software lamp (Sankararaman et al., 2008) followed by elai (Guan, 2014). lamp allows ancestry block estimation without defining a priori ancestral nonadmixed populations. This unique feature was required for the analysis of our dataset, because most Eurasian wolf populations include individuals showing signature of past admixture with dogs (Fan et al., 2016; Pilot et al., 2018), and some FRD populations and dog breeds show evidence of admixture with wolves (Kopaliani et al., 2014; Pilot et al., 2015). In lamp, identification of ancestral populations was an integrated part of the admixture analysis.

In the lamp analysis, we assumed a mixture proportion of 0.33:0.67, which was determined based on the frequency of wolves versus dogs in the dataset (506 vs. 1020 individuals). This ratio accommodated a conservative scenario where none of the individuals is admixed. We used a recombination rate of 1 × 10^−10^ per base pair per generation and fraction of overlap between adjacent windows (offset) of 0.2. We assumed 10 generations since admixture, because power to detect recent admixture was diminished when more distant admixture events were assumed (Pilot et al., 2018).

Based on the lamp results, we identified individuals that were not admixed or had very low levels of inferred admixture. These individuals were used to define reference genotype sets for wolves and dogs, which are required by the elai analysis to estimate allele frequencies in nonadmixed populations. Thresholds to classify individuals as nonadmixed were set at 0.001 for dogs and 0.01 for wolves. The different thresholds account for different distributions of admixture proportions in wolves and dogs and were established so as to obtain at least 300 individuals in each nonadmixed reference sets. Due to the use of these thresholds, a small number of introgressed fragments present in the reference genotypes were considered as native and thus could not be identified as introgressed blocks in the individuals tested using elai, potentially leading to the underestimation of admixture. The alternative of including in the reference genotype sets only individuals that were identified in lamp as having no introgressed fragments would result in the reference genotype sets being smaller and not representative of all regional genetic variation (i.e., some wolf populations would not be represented and the dog reference set would consist mostly of pure‐bred dogs). Using such reference sets could have resulted in the overestimation of admixture, and therefore we used the first, more conservative approach.

The resulting reference datasets included 304 nonadmixed dogs and 334 nonadmixed wolves, representing all primary geographic populations and breed groups studied. The remaining 717 dogs and 171 wolves (888 individuals), which were not included in the reference genotype sets, were further tested for admixture using elai. Although we used the set on nonadmixed individuals identified in lamp as input for the elai analysis, these analyses were otherwise independent. If lamp incorrectly inferred nonexistent admixture, it was possible for elai to identify all individuals as pure wolves and dogs. Accordingly, if lamp underestimated admixture, it was possible for elai to identify considerably higher admixture proportions compared with lamp.

We used 20 expected maximization steps to estimate the parameters of the hidden Markov model implemented in elai. elai accounts for the possibility of continuous admixture throughout multiple generations, enabling a more realistic representation of wolf‐dog admixture. Unlike lamp, it does not require filtering for LD, and thus, ancestry could be inferred for all SNPs in the initial dataset (106,549 autosomal SNPs). elai can detect ancestry blocks <1 cM and accounts for the presence of population substructure within each of the admixing entities. In our analysis, we assumed admixture between two main population clusters (wolves vs. dogs) during 100 generations, and the presence of 10 lower‐layer clusters (advised to be five times the number of upper‐level clusters).

Further, elai does not require phasing or a recombination map, because it directly estimates cluster‐switch rates between adjacent markers, which enables the direct inference of recombination rates at each locus from the data analysed (Guan, 2014). There is considerable variation in recombination rates between sexes and between individuals within species (e.g., Kong et al., 2010), and therefore, even a high‐resolution recombination map will not necessarily be accurate if the analysed dataset is considerably different than the dataset used to construct the map. Hence, we selected elai as the preferred software for the admixture analysis.

### 2.3 Assessment of the effect of recombination rate on estimated admixture proportions

To assess whether the variation in local admixture proportions across the genome (inferred using elai) is dependent on recombination rates, we tested for the correlation between these two variables, first for all SNP loci across 38 autosomal chromosomes and then for each chromosome separately. We used the average recombination rates for males and females provided by Campbell et al. (2016). We selected the recombination map from this study over other available high‐density maps (Auton et al., 2013; Axelsson et al., 2012), because (a) this map was constructed using the Illumina CanineHD Whole‐Genome Genotyping BeadChip, which was the same array as in our study, and (b) it is a pedigree‐based map, which is expected to be more accurate than LD‐based maps that can be affected by demographic patterns specific to the study populations. We used R 4.0.3 (R Core Team, 2020) to calculate Pearson's correlation coefficient and to fit a linear regression model to the data.

### 2.4 Detection of chromosomal fragments with overrepresentation of introgressed variants

The elai analysis included only 717 dogs and 171 wolves for which the admixture proportions estimated in lamp were above the established thresholds. Pure‐bred dogs were excluded from further analysis, as we were interested in natural introgression patterns only. Both East Asian and North American wolves were also excluded due to small numbers of admixed individuals detected (four in each population). We excluded Mexican wolves as well, even though many individuals carried a small proportion of dog admixture, since this population is highly inbred (Fredrickson et al., 2007), and admixture may have resulted from a single event, possibly during captive breeding.

Analyses of introgression patterns focused on the remaining set of admixed individuals, which comprised 88 West Eurasian wolves and 201 Eurasian FRDs (Figure 1). Based on elai results, we calculated the mean admixture proportions within each autosomal chromosome and across autosomal chromosomes in both datasets. We identified chromosomal blocks with hybrid ancestry either greater or smaller than three standard deviations (SD) from the mean for each chromosome (see Figure S1), thus permitting identification of blocks with overrepresented or underrepresented hybrid ancestry, respectively. To reduce the false‐positive rate, we only considered ancestry blocks that included at least 10 sequential SNPs.

Blocks with overrepresented or underrepresented hybrid ancestry were identified based on the SD at the level of individual chromosomes rather than the global SD across all autosomal loci, because chromosomes are natural genetic units with independent recombination. This approach is consistent with the previous step of the study, that is the detection of admixture and reconstruction of the distribution of introgressed blocks, which was done at the level of individual chromosomes. Accordingly, the detection of selection signatures was also based on patterns of extended haplotype homozygosity within individual chromosomes (see below), so our approaches for all the consecutive analyses were consistent. For comparative purposes, we also carried out the identification of overrepresented or underrepresented hybrid ancestry based on the global SD estimates, which is reported in the Supplementary Materials.

To estimate the false‐positive and false‐negative rate associated with the detection of blocks with overrepresented hybrid ancestry, we applied a random resampling approach. We randomly selected 38 chromosomal blocks by choosing a position within a selected autosomal chromosome (both determined randomly). A number of consecutive SNPs to be included within the block (counting from the selected position) was also chosen randomly from a range of 10 to 150. These 38 blocks were then assembled into a “randomised chromosome.” This was done separately for wolves and FRDs. The “randomised chromosome” is representative of autosomal chromosomes from their population of origin and reflects the population's admixture proportions as well as the demographic processes and evolutionary forces that shaped the gene pool, but is free from chromosome‐specific recombination patterns that could result from incomplete sampling of the parental population genetic diversity. We analysed introgression patterns in these “randomised chromosomes” in the same way as real chromosomes in order to identify chromosomal blocks with significantly overrepresented hybrid ancestry. We then estimated error rates by assessing whether (a) blocks with significantly overrepresented hybrid ancestry identified within a “randomised chromosome” were identified as having no overrepresented hybrid ancestry in the real chromosomes (false‐negative rate), and (b) blocks with no significantly overrepresented hybrid ancestry in the context of a “randomised chromosome” were identified as having overrepresented hybrid ancestry in the context of their real chromosomes (false‐positive rate).

### 2.5 Identification of loci under positive selection

The genotype data for each chromosome that contained ancestry blocks showing overrepresentation of introgressed alleles were phased using fastPHASE (Scheet & Stephens, 2006). For the phased data, we performed scans for signatures of selection across autosomal chromosomes using the Integrated Haplotype Homozygosity Score (iHS) statistics (Voight et al., 2006), as implemented in the R package *rehh v.3.1.2* (Gautier et al., 2017). The iHS statistics is derived from the extended haplotype homozygosity (EHH) statistics, which measures homozygosity decay in haplotypes carrying a specified “core” SNP at one end, with increasing haplotype length (Sabeti et al., 2002). An allele that rises rapidly in frequency due to selection will have high levels of haplotype homozygosity extending over a larger distance than expected under a neutral model (Sabeti et al., 2002). The Integrated Haplotype Homozygosity Score (iHS) is based on the integral of the observed EHH decay away from a specified core SNP until it reaches the value of 0.05 (Voight et al., 2006). We used the *rehh* package's functions *scan_hh* and *ihh2ihs* to calculate the iHS statistics and its two‐sided *p*‐value for each SNP. Phasing and *rehh* analysis were conducted separately for wolf and FRD genotypes.

Introgression can change the distribution of allele frequencies and haplotype structure, which can potentially interfere with the detection of selection signatures using iHS (Booker et al., 2017). However, while introgression can distort haplotype structure in any part of the genome, selection can only act on those parts of the genome that have a biological function (including protein‐coding genes, long noncoding RNA and regulatory elements) and DNA regions in local proximity that may be affected by selective sweeps. Therefore, we assessed the error rate associated with the detection of adaptive introgression as the proportion of SNPs that are inferred to be under selection, but are located a sufficient distance from protein‐coding genes and long noncoding RNA that they are unlikely to be affected by a selective sweep. We assumed this distance to be 100 kb, and functional information was based on the Ensemble CanFam3.1 dog genome annotation.

We considered the iHS results as significant if *p* < 0.05 and |iHS| > 2 and did not use a correction for multiple testing. The study that introduced the iHS statistics showed that |iHS| >2 is a powerful criterion to identify signals of selection and did not use or recommend corrections for multiple testing (Voight et al., 2006). Here, we focused on signatures of selection within relatively short chromosomal blocks (161–4493 kb) showing overrepresentation of introgressed variants and did not attempt to identify loci showing signatures of selection across the entire genome. The signal of selection is not independent for individual SNPs, given that the iHS test is based on haplotype homozygosity. Therefore, loci showing signatures of selection are expected to be clustered (e.g., we found up to 14 significant SNPs within one gene). For these reasons, we report candidate genes identified using this test without correcting for multiple testing. However, in order to show how correction for multiple testing could affect our interpretation, we also report the candidate genes identified after applying the Bonferroni correction, based on the number of SNPs within each chromosome that were used to obtain the iHS statistics.

### 2.6 Gene ontology enrichment analysis

Ensembl was used to identify coding genes located within chromosomal blocks displaying overrepresentation of hybridization‐derived variants, based on CanFam3.1 dog genome assembly. We considered a gene as being located within a chromosomal block if the entire open reading frame or only a part of the reading frame was located within that block. We also generated an additional set of genes of interest by identifying human genes orthologous to canine genes found within those blocks using synteny analysis. The two gene sets were kept separate for further analyses. As each gene set was created separately for wolves and FRDs, we generated a total of four gene sets.

Each gene set was tested for Gene Ontology (GO) term enrichment using the web‐based software g:Profiler (Raudvere et al., 2019). This analysis was carried out separately for wolves and FRDs. Genes identified based on the dog genome assembly were compared to the reference set of annotated genes from the dog genome. Human orthologues were compared to the reference set of annotated genes from the human genome assembly (GRCh38.p13). A significance threshold of 0.05 with Benjamini–Hochberg correction was used, as well as the more conservative g:SCS (Set Counts and Sizes) false discovery rate correction method that accounts for multiple testing due to the overlap of functional terms (Reimand et al., 2007).

## 3 RESULTS

### 3.1 Admixture proportions in Eurasian wolves and free‐ranging dogs

Genetic differentiation between Eurasian wolf and free‐ranging dog populations was estimated at *F*_ST_ = 0.205. The lamp analysis estimated genome‐wide admixture proportions of 0.0075 in Eurasian FRDs, 0.0274 in West Eurasian wolves, 0.0031 in East Asian wolves and 0.0047 in North American wolves (excluding Mexican wolves). Assuming a 1% admixture threshold, 66% of Western Eurasian wolves, 8% of East Asian wolves and 2% of North American wolves studied were identified as admixed.

The elai analysis was consistent with lamp in the identification of admixed individuals. The proportion of dog admixture in 88 admixed West Eurasian wolves estimated by ELAI ranged from 0.010 to 0.263 (an average of 0.041). The wolf admixture proportion in 201 admixed Eurasian FRDs was lower, ranging from 0.001 to 0.101 (an average of 0.023).

We found differences in the frequency of individuals carrying introgressed ancestry blocks between geographic regions in both wolves and FRDs. In the case of wolves, the highest frequency of such individuals was found in Europe, lower in the Caucasus and Mongolia and the lowest in Yakutia. In the case of FRDs, high frequency of dogs carrying hybridization‐derived variants was found in several geographically distinct regions, including Saudi Arabia, Mongolia and South‐East Asia (Figure S2).

### 3.2 The effect of the recombination rate on admixture proportions

In the regression models fitted to the set of SNPs from across all autosomal chromosomes, we found no significant correlation between local admixture proportions and recombination rates in either wolves (Pearson's *R* = −0.004, 11,224 *df*, *p* = 0.6874) or dogs (*R* = 0.010, 11,224 *df*, *p* = 0.3117). At the level of individual chromosomes, no consistent correlation pattern was found between the local admixture proportions and recombination rates in either wolves or dogs. The correlation was nonsignificant for most chromosomes (31 in wolves, 36 in dogs), and both positive and negative significant correlations were observed in the remaining chromosomes in both canids (Table S2).

We also assessed the correlation between the local admixture proportions in wolves versus dogs. If recombination had a significant effect on admixture proportions, the same effect should be expected in wolves and dogs, resulting in a positive correlation between their local admixture proportions. Instead, we found a weak, but significant negative Pearson's correlation (*R* = −0.042, 11,224 *df*, *p* = 7.384 × 10^−6^) between the local admixture proportions in wolves versus dogs. The chromosome‐level analysis did not reveal a consistent pattern: a significant negative correlation between local admixture proportions was found for 13 chromosomes, a significant positive correlation for 11 chromosomes and no significant correlation for the remaining 14 chromosomes (Table S2).

### 3.3 Chromosomal blocks with significantly overrepresented hybridization‐derived ancestry

Although average genome‐wide admixture proportion was higher in West Eurasian wolves than Eurasian FRDs, the opposite pattern was observed with regard to the number and length of chromosomal blocks with significantly overrepresented hybridization‐derived ancestry. This was only the case when outlier blocks were identified at the level of individual chromosomes; when the standard deviation was calculated across all autosomal chromosomes, a larger number of significantly overrepresented introgressed blocks was identified in wolves than in dogs (Table S3). This result was, however, biased by the presence of several regions in the dog genome with wolf admixture proportions 5–15 times higher than the global mean. As a result, the global standard deviation across all loci (0.23) was considerably higher than the mean standard deviation across 38 within‐chromosome means (0.17). This prevented the detection of local outliers, with the exception of those few with extreme values that reached the global threshold. Therefore, we report the results of outlier block identification based on the global standard deviation in the Supplementary Materials (Table S3, Figures S3 and S4), while the results reported below are based on the detection of outlier blocks within autosomal chromosomes. It should be noted that we did not analyse the admixture patterns within the X chromosome, because our dataset did not include X chromosome SNPs (see Section 2.1).

**FIGURE 2:**
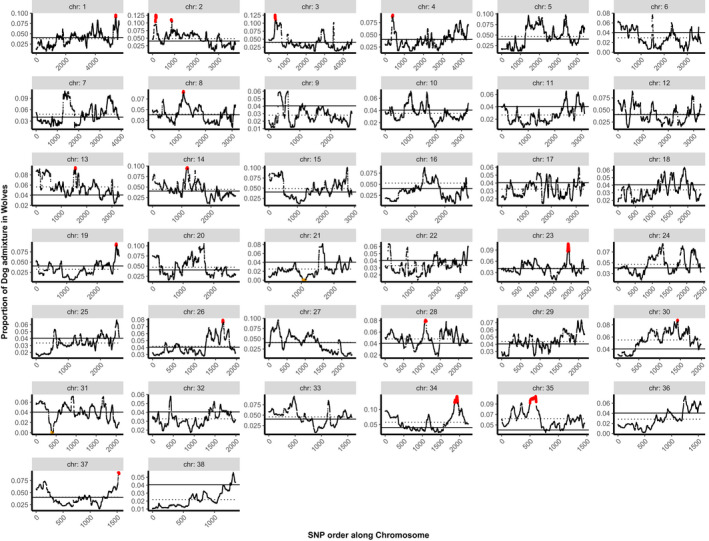
Distribution of dog ancestry in admixed West Eurasian wolves. *x*‐axis shows SNP order along each autosomal chromosomes (without reflecting physical distances between SNP loci), and *y*‐axis shows the proportion of dog admixture in wolves (with only admixed individuals considered). The solid horizontal line represents the mean dog admixture across autosomal chromosomes, and the dotted horizontal line represents the mean dog admixture within each chromosome. Chromosomal blocks with overrepresented dog ancestry are marked in red and are defined as having at least 10 sequential SNPs with the proportion of dog ancestry >3 SD above the mean, which was assessed at the level of individual chromosomes. Ancestry deserts are marked in orange

**FIGURE 3:**
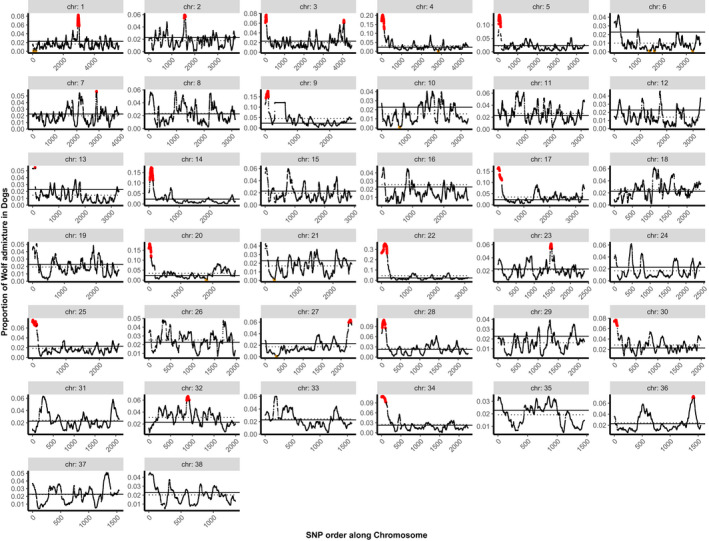
Distribution of wolf ancestry in admixed Eurasian free‐ranging dogs (FRDs). *x*‐axis shows SNP order along each autosomal chromosomes (without reflecting physical distances between SNP loci), and *y*‐axis shows the proportion of wolf admixture in FRDs (with only admixed individuals considered). The solid horizontal line represents the mean wolf admixture across autosomal chromosomes, and the dotted horizontal line represents the mean wolf admixture within each chromosome. Chromosomal blocks with overrepresented wolf ancestry are marked in red and are defined as having at least 10 sequential SNPs with the proportion of wolf ancestry >3 SD above the mean, which was assessed at the level of individual chromosomes. Ancestry deserts are marked in orange

In West Eurasian wolves, we identified 16 blocks with overrepresented dog ancestry on 15 chromosomes (Table 1, Figure 2), with an average dog ancestry per block between 0.078 and 0.131, and a global average across all blocks of 0.098. In FRDs, we identified 21 blocks with overrepresented wolf ancestry on 20 chromosomes (Table 1, Figure 3), with an average proportion of wolf ancestry per block between 0.055 and 0.315, and a global average of 0.106. The average block size was 819 Kb in wolves and 1919 Kb in FRDs. The set of coding genes located within these blocks (further referred to as “OHA genes” to reflect their location within the overrepresented hybrid ancestry blocks) included 61 genes in wolves and 294 in FRDs. Synteny analysis with the human genome identified larger numbers: 72 in wolves and 311 in FRDs. The chromosomal blocks discussed above do not include seven short blocks (4–116 Kb) for which the number of SNPs ranges between two and nine. These were removed from the analysis to reduce the number of false positives. Three of these blocks included one coding gene each, while the remaining blocks did not include any.

**TABLE 1:** Chromosomal blocks with overrepresentation of introgressed ancestry in wolves and FRDs

Population	chr	Chromosomal block position	Block size	Average introgressed ancestry	*N* genes	*N* SNP loci	*N* CAI genes	*N* CAI SNP loci
Wolves	1	1‐118527129 ‐ 1‐118918741	391,612	0.094	3/3	26	0	0
Wolves	2	2‐3579904 ‐ 2‐7247465	3,667,561	0.121	8/13	44	2	2
Wolves	2	2‐21701739 ‐ 2‐22475191	773,452	0.110	6/6	33	1	1
Wolves	3	3‐9217526 ‐ 3‐9862481	644,955	0.118	1/1	36	0	0
Wolves	4	4‐7674062 ‐ 4‐8250087	576,025	0.089	7/8	30	0	0
Wolves	8	8‐27548538 ‐ 8‐28277904	729,366	0.083	4/4	24	0	0
Wolves	13	13‐28216740 ‐ 13‐28398676	181,936	0.093	1/1	13	0	0
Wolves	14	14‐27898988 ‐ 14‐28678482	779,494	0.095	1/1	40	0	0
Wolves	19	19‐52067458 ‐ 19‐52340491	273,033	0.093	1/3	21	0	0
Wolves	23	23‐43608851 ‐ 23‐44248615	639,764	0.098	9/9	41	0	0
Wolves	26	26‐34215853 ‐ 26‐34425449	209,596	0.078	1/1	16	0	0
Wolves	28	28‐20956513 ‐ 28‐21517197	560,684	0.080	2/2	37	0	0
Wolves	30	30‐29515850 ‐ 30‐29677034	161,184	0.087	2/2	10	0	0
Wolves	34	34‐35623963 ‐ 34‐37106724	1,482,761	0.131	8/9	83	1^a^	1
Wolves	35	35‐8750539 ‐ 35‐10496444	1,745,905	0.100	5/7	109	0	0
Wolves	37	37‐30592158 ‐ 37‐30874615	282,457	0.091	2/2	17	0	0
Wolves	Mean		818,737	0.098	3.8/4.5	36.3	0.3	0.3
FRDs	1	1‐60419833 ‐ 1‐62667942	2,248,109	0.073	8/8	95	6	9
FRDs	2	2‐35398496 ‐ 2‐37126070	1,727,574	0.058	42/43	54	3	4
FRDs	3	3‐418639 ‐ 3‐2094780	1,676,141	0.072	8/8	57	1	1
FRDs	3	3‐85336069 ‐ 3‐85680942	344,873	0.064	3/3	23	0	0
FRDs	4	4‐43598 ‐ 4‐3426340	3,382,742	0.181	12/13	133	2	8
FRDs	5	5‐1559873 ‐ 5‐3379769	1,819,896	0.127	2/2	97	2	4
FRDs	7	7‐62475370 ‐ 7‐62647106	171,736	0.056	1/1	10	0	0
FRDs	9	9‐779889 ‐ 9‐2719467	1,939,578	0.162	30/30	76	10	20
FRDs	13	13‐2213820 ‐ 13‐2419264	205,444	0.055	1/1	10	0	0
FRDs	14	14‐2529729 ‐ 14‐4675587	2,145,858	0.154	14/14	99	4	10
FRDs	17	17‐51048 ‐ 17‐3686421	3,635,373	0.138	17/18	150	8	11
FRDs	20	20‐144948 ‐ 20‐3406396	3,261,448	0.164	32/38	72	3	3
FRDs	22	22‐108262 ‐ 22‐4593585	4,485,323	0.315	32/32	196	16	19
FRDs	23	23‐33728739 ‐ 23‐34485710	756,971	0.059	7/7	42	0	0
FRDs	25	25‐151268 ‐ 25‐4644607	4,493,339	0.070	24/25	129	8	26
FRDs	27	27‐44109742 ‐ 27‐44912153	802,411	0.062	8/8	60	1	14
FRDs	28	28‐488365 ‐ 28‐2383854	1,895,489	0.101	22/24	84	6	12
FRDs	30	30‐1366 ‐ 30‐1819062	1,817,696	0.073	21/26	70	5	9
FRDs	32	32‐16465021 ‐ 32‐17467739	1,002,718	0.062	6/6	60	1	1
FRDs	34	34‐127067 ‐ 34‐2342162	2,215,095	0.104	4/4	115	2	10
FRDs	36	36‐28437436 ‐ 36‐28698900	261,464	0.071	0/0	22	0	0
FRDs	Mean		1,918,537	0.106	14.0/14.9	78.8	3.7	7.7

Note

*N* genes—number of genes within the chromosomal block; two values reported represent *N* genes annotated in the canine genome/*N* orthologous genes annotated in the human genome identified in the synteny analysis. *N* SNPs—number of genotyped SNPs within the chromosomal block. *N* CAI genes—number of candidate genes subject to adaptive introgression. *N* CAI SNPs—number of genotyped SNP loci putatively subject to adaptive introgression.

^a^ The gene is located 6Kb downstream of the SNP showing signature of positive selection.

We also used the lamp results to look at chromosomal blocks featuring underrepresentation of hybrid ancestry, using the analogous criterion as for overrepresentation of hybrid ancestry, that is frequency of hybridization‐derived variants less than three SD below the mean. We did not find chromosomal blocks meeting this criterion in either wolves or FRDs, possibly because the threshold applied corresponded to strict “ancestry deserts,” that is regions with no hybrid ancestry. When we applied a criterion of <0.1% of hybrid ancestry (as in Sankararaman et al., 2014), we identified two blocks with underrepresented dog ancestry in wolves and ten blocks with underrepresented wolf ancestry in FRDs (Table S4).

The error rate estimate based on the “randomised chromosomes” showed that in both wolves and FRDs, one out of the 38 randomly selected blocks was identified as a significant outlier, even though it was not significant in the original analysis (Figure S5). This gives a false‐positive rate of 3%. In FRDs, one chromosomal block that was identified as a significant outlier in the original analysis was not significant in the context of the “randomised chromosome,” thus resulting in a false‐negative rate of 3%. In wolves, we found no false negatives, but we found one chromosomal block that was identified as a significant outlier in both the original analysis and in the context of the “randomised chromosome” (true positive). The remaining 36 blocks were identified as true negatives in both wolves and FRDs. The false‐positive rate was therefore 3% and the average false‐negative rate was 1.5%.

### 3.4 Functional characterization of the OHA genes

We carried out a GO enrichment analysis on the OHA gene sets for both wolf and FRD populations. Due to differences in annotation, the resulting set of overrepresented GO terms differed between the canine genes and their human orthologues (Table S5). The GO analysis for the OHA gene set in wolves, based on the canine assembly, showed overrepresentation of the molecular function “ATP‐dependent peptidase activity.” The analysis based on human homologues revealed overrepresentation of biological processes “amino acid neurotransmitter reuptake” and “glutamate reuptake.” All these terms remained overrepresented with both the Benjamini–Hochberg correction and the more strict g:SCS correction.

The OHA gene set in FRDs contained a larger set of enriched GO terms compared to that observed in wolves (Table S5). Terms “homophilic cell adhesion via plasma membrane adhesion molecules” and “cell‐cell adhesion via plasma‐membrane adhesion molecules” were identified as overrepresented in the analyses based on both canine genes and human homologues and with both corrections. These GO terms are specific to clustered protocadherins (*PCDH*), which are a subset of genes from the protocadherin family clustered in a single chromosomal block in vertebrate genomes (Chen & Maniatis, 2013). In the FRDs studied, the block located on chromosome 2 had a significantly higher proportion of alleles introgressed from wolves compared to the whole‐genome average and included 13 annotated *PCDH* genes, with additional coding genes identified but not annotated. The OHA gene set in wolves included an additional protocadherin gene, *PCDH15*, which is located on a different chromosome (chr 26) than the clustered protocadherins.

Other overrepresented GO terms in the OHA gene set for FRDs described more general biological processes involving *PCDH* genes: “cell‐cell adhesion,” “cell adhesion,” “biological adhesion.” Among the molecular functions overrepresented in this gene set were “cation binding,” “metal ion binding” and “calcium ion binding,” which are common functions of *PCDH* genes and several other unlinked genes (Table S5).

The OHA gene set for FRDs also showed overrepresentation of the molecular function “olfactory receptor activity,” which was a common function of 17 olfactory receptor (*OR*) genes identified in an analysis based on human homologues (with eight other *OR* genes excluded from the GO analysis by the software). These genes were located on three distinct chromosomes. The “olfactory receptor activity” was significantly enriched only when Benjamini–Hochberg correction was used and should therefore be considered with caution. In the GO analysis focused on canine genes, this term did not show significant overrepresentation (with 11 *OR* genes included), which is likely because the *OR* gene annotation is less complete in the dog genome assembly and is known to represent many pseudogenes.

### 3.5 Candidate genes under positive selection within introgressed chromosomal blocks

Using the iHS statistics, we found 324 SNPs showing signatures of selection within chromosomal blocks with significant overrepresentation of hybridization‐derived ancestry in FRDs. Based on the Ensemble CanFam3.1 dog genome annotation, we found that 153 SNPs (47.2%) were located within protein‐coding genes (Table S6), 16 SNPs (4.9%) within long noncoding RNA, and 126 SNPs (38.9%) were located within 100 kb from protein‐coding genes or long noncoding RNA. The remaining 33 SNPs (10.2%) were outside the 100 kb window from any annotated gene. In this case, we can therefore infer a false discovery rate of 10.2% associated with the detection of selection signatures. In wolves, we found only 18 SNPs showing signatures of selection within blocks with overrepresentation of hybrid ancestry, four (22.2%) of which were located within protein‐coding genes (Table S6) and 12 (66.7%) within 100 kb from protein‐coding genes. Two (11.1%) were more than 100 kb from any gene, which gives a false discovery rate of 11.1%, similar to the rate estimated in FRDs. We note that this error rate is a conservative estimate, given that functional annotation in the dog genome is less complete compared with the human genome (e.g., see the results on the Gene Ontology analysis in this study) and the function of some constrained elements in mammalian genomes remains unknown. A comparative study of 29 mammalian genome sequences showed that about 55% of the constrained elements were located within coding genes (exons, UTRs and introns), 4.4% within 2 kb of transcriptional start sites, 1.5% within RNA genes and 38.6% in intergenic regions. This implies that about 38.6% of constrained elements could not be assigned a function at the time (Lindblad‐Toh et al., 2011).

If one or more SNPs showing signatures of positive selection were located within a protein‐coding gene, such gene was considered as a candidate gene under positive selection. Such genes constituted 23% of the OHA genes in FRDs and 5.6% in grey wolves. Genes showing signatures of positive selection that are located within chromosomal blocks showing significant overrepresentation of hybridization‐derived ancestry, are strong candidates for adaptive introgression and therefore will be referred to as “CAI genes” hereafter. In this study, we did not define the functional variants in the CAI genes and therefore we cannot infer the exact phenotypic effect of introgression of wolf‐derived variants into FRDs, or in the reverse direction. However, the GO analysis and the data from published studies regarding the function of the CAI genes provide information on the general type of phenotypic traits that may be subject to adaptive introgression.

In grey wolves, we identified three positively selected SNPs within three genes from the set of 72 OHA genes (Table S6). These three genes (*ABI1*, *APBB1IP* and *FRMD4A*) were located within two OHA blocks set 14.5 Mb apart on chromosome 2. Another SNP under positive selection was identified six Kb upstream of *PLD1* gene on chromosome 34. Within this set of four CAI genes in wolves, the GO terms for two cellular components, “cell junction” and “lamellipodium”—a cytoskeletal projection on the leading edge of motile cells—were overrepresented, with all four genes contributing to the first term and two genes contributing to the second. These two terms were identified as overrepresented only in the analysis involving human homologues of canine genes, while the analysis based on canine genes gave no significant results.

Free‐ranging domestic dogs had a much larger set of CAI genes. Seventy‐two of 311 OHA genes (23%) in FRDs, distributed throughout 16 chromosomal blocks, contained SNPs subject to positive selection (Table 1, Table S6). The highest number of SNP under selection per gene was found in the *CACNA1C* gene, with all 14 SNPs genotyped within this gene showing signatures of selection based on the *rehh* analysis.

In the set of 72 CAI genes in FRDs, the majority of overrepresented GO terms were associated with calcium channel activity, with several genes (*CACNA1C*, *RYR2*, *RYR3*, *TRPC4*, *TRDN*, *ATP7B*, *HTR2A*) contributing to multiple terms (Table 2). Most of these genes (*CACNA1C*, *RYR3*, *TRPC4*, *HTR2A*) are expressed in the brain and associated with neurodevelopment, behavioural traits and cognitive functions (Table 3). In addition, *CACNA1C* and *HTR2A*‐encoded proteins are involved in viral infections, acting as receptors for influenza virus and JC polyomavirus, respectively (Assetta et al., 2013; Fujioka et al., 2018). RYR2 and TRDN proteins are functionally linked and constitute the main component of a calcium channel that supplies ions to the cardiac muscle enabling its contraction (Györke et al., 2004).

**TABLE 2:** Candidate genes under adaptive introgression in grey wolves (“wolves”) and free‐ranging domestic dogs (“FRDs”) that contributed to enriched GO terms

Population	chr	Gene	*N* SNPs	Enriched GO terms
Wolves	2	ABI1	1	Cell junction, lamellipodium
Wolves	2	APBB1IP	1	Cell junction, lamellipodium
Wolves	2	FRMD4A	1	Cell junction
Wolves	34	PLD1	1	Cell junction
FRDs	1	TRDN	1	Maintenance of location in cell, terms associated with calcium channel
FRDs	4	RYR2	7	Ryanodine‐sensitive calcium‐release channel activity, other terms associated with calcium channel, cell–cell signalling involved in cardiac conduction and related terms
FRDs	22	HTR2A	1	Maintenance of location in cell, sequestering of calcium ion
FRDS	22	ATP7B	2	Maintenance of location in cell, cellular ion homeostasis and related terms
FRDS	25	TRPC4	2	Calcium channel activity and related terms
FRDS	27	CACNA1C	14	Maintenance of location in cell, terms associated with calcium channel, cell–cell signalling involved in cardiac conduction and related terms
FRDs	30	RYR3	5	Ryanodine‐sensitive calcium‐release channel activity, other terms associated with calcium channel
FRDs	9	GAA	1	Axoneme assembly and related terms, maintenance of location in cell
FRDs	9	DNAH17	1	Axoneme assembly and related terms
FRDs	14	LRGUK	3	Axoneme assembly and related terms
FRDs	34	DNAH5	6	Axoneme assembly and related terms
FRDs	17	FAM110C	1	Cell projection assembly
FRDs	17	SH3YL1	2	Cell projection assembly
FRDs	9	CYTH1	2	ARF guanyl‐nucleotide exchange factor activity
FRDs	20	IQSEC1	1	ARF guanyl‐nucleotide exchange factor activity
FRDs	9	RPTOR	5	Protein serine/threonine kinase inhibitor activity
FRDs	28	NCOA4	1	Cellular ion homeostasis and related terms
FRDs	30	SLC12A6	1	Metal ion transmembrane transporter activity

Note

*N* SNPs—the number of SNPs within the gene with signatures of positive selection inferred using *rehh*. For the complete list of candidate genes under adaptive introgression, see Table S5.

**TABLE 3:** Functions of candidate genes under adaptive introgression in free‐ranging domestic dogs (“FRDs”) and grey wolves (“wolves”) associated with the common groups of enriched GO terms (e.g., “calcium channel” denotes all GO terms related to calcium channel activity)

Population	Gene	GO terms	Function
Wolves	ABI1	Cell junction	Plays an essential role in synapse formation (Proepper et al., 2007).
Wolves	APBB1IP	Cell junction	Associated with schizophrenia and prepulse inhibition (Ashbrook et al., 2019).
Wolves	FRMD4A	Cell junction	Associated with congenital microcephaly and Alzheimer's disease (Fine et al., 2015; Lambert et al., 2013).
Wolves	PLD1	Cell junction	Plays a key role in neurotransmitter release and regulates dendrite morphogenesis (Zhu et al., 2012).
FRDs	TRDN	Calcium channel	Physically links the RYR2 and CASQ2 proteins, enabling the regulation of RYR2 channel activity by CASQ2 (Györke et al., 2004).
FRDs	RYR2	Calcium channel	Ryanodine receptor, which forms intracellular calcium channels in excitable tissues such as muscles and neurons (Santulli & Marks, 2015). Primarily expressed in heart muscle, but affects also neurobiological processes; variation in this gene is responsible for sex differences in autism (Chen et al., 2017).
FRDs	HTR2A	Calcium channel	Encodes one of the serotonin receptors, which is a target of serotonergic psychedelic drugs and antipsychotic drugs (Moreno et al., 2011) that plays a role in learning and memory (Harvey, 2003) and is a receptor for JC polyomavirus (Assetta et al., 2013). Regulates levels of several hormones—oxytocin, ACTH, corticosterone, renin and prolactin (Van de Kar et al., 2001).
FRDs	ATP7B	Calcium channel	Copper transmembrane transporter (Braiterman et al., 2014)
FRDs	TRPC4	Calcium channel	Forms a calcium‐permeable cation channel, which plays a role in multiple processes including neurotransmitter release. Expressed in midbrain dopamine neurons and affects behavioural traits such as attention span and sociability (Illig et al., 2011; Rasmus et al., 2011). Involved in lung endothelial permeability (Tiruppathi et al., 2002).
FRDs	CACNA1C	Calcium channel	Encodes an L‐type calcium channel Ca_v_1.2, which is a critical mediator of brain development and experience‐dependent brain plasticity. One of the most widely reproduced candidate genes for multiple neuropsychiatric disorders. Affects social behaviour and cognitive function (Kabir et al., 2017). Ca_v_1.2 is a receptor for influenza virus (Fujioka et al., 2018).
FRDs	RYR3	Calcium channel	Ryanodine receptor, which forms intracellular calcium channels in excitable tissues such as muscles and neurons (Santulli & Marks, 2015). Expressed in a broad range of tissues, including the brain, where it affects synaptic plasticity (Balschun et al., 1999).
FRDs	GAA and CCDC40	Axoneme assembly	*GAA* and *CCDC40* genes are located directly next to each other in mammalian genomes, with known insertions–deletions encompassing both genes (Amiñoso et al., 2013). CCDC40 regulates the assembly of the inner dynein arm and the dynein regulatory complexes and thus is essential for correct functioning of motile cilia (Becker‐Heck et al., 2011).
FRDs	DNAH17	Axoneme assembly	Axonemal dynein—a cytoskeletal motor protein that moves along microtubules, driving the beat of eukaryotic cilia and flagella (King, 2018). A sperm‐specific dynein associated with reduced sperm motility (Whitfield et al., 2019).
FRDs	LRGUK	Axoneme assembly	Involved in the early stages of axoneme development and in multiple aspects of sperm assembly including sperm head shaping (Liu et al., 2015).
FRDs	DNAH5	Axoneme assembly	Axonemal dynein—a cytoskeletal motor protein that moves along microtubules, driving the beat of eukaryotic cilia and flagella (King, 2018). Is expressed in lungs and associated with respiratory ciliary disorders (Fliegauf et al., 2005).

Another set of overrepresented terms was associated with the axoneme, which is the main cytoskeletal structural component of a cilium or flagellum (Ishikawa, 2017), with *DNAH5*, *DNAH17*, *GAA‐CCDC40* and *LRGUK* genes contributing to these terms (Tables 2 and 3). Formation of ciliary axonemal structures is necessary for regulating motility and beating of the cilia. Therefore, mutations in genes associated with axonemal architecture frequently lead to primary ciliary dyskinesia, characterized by recurrent infections of the respiratory tract and sperm immobility (Lee & Gleeson, 2011; Olbrich et al., 2002).

As explained in the Methods, candidate genes listed above were identified without correction for multiple testing. After applying a Bonferroni correction based on the number of SNPs tested for each chromosome, we found no loci under positive selection within chromosomal blocks with overrepresented hybrid ancestry in wolves and only two such loci in FRDs. These were located within the serotonin receptor subtype 2A (*HTR2A*) and the type 3 ryanodine receptor (*RYR3*) genes. *HTR2A* encodes one of the serotonin receptors, which plays a role in learning and memory (Harvey, 2003). *HTR2A* is expressed widely in the brain and regulates levels of several hormones—oxytocin, ACTH, corticosterone, renin and prolactin (Van de Kar et al., 2001). RYR3 protein forms intracellular calcium channels in excitable tissues such as muscles and neurons (Santulli & Marks, 2015). It is expressed in a broad range of tissues, including the brain, where it affects synaptic plasticity (Balschun et al., 1999). The two strongest candidate genes for adaptive introgression in FRDs are thus well‐described genes affecting neurobiological processes.

## 4 DISCUSSION

### 4.1 Factors affecting introgression patterns in wolves and free‐ranging dogs

Introgression from dogs has an important effect on the gene pool composition of Western Eurasian wolf population, with the average of 2.7% dog admixture. By comparison, the average Neanderthal admixture proportion in modern humans in Eurasia is estimated at 2% and is thought to have an important effect on multiple human phenotypic traits as well as on overall fitness (Harris & Nielsen, 2016; Racimo et al., 2017). East Asian and North American wolf populations have considerably lower admixture proportions than Western Eurasian wolves (0.3% and 0.5%, respectively). In Eurasia, the highest frequency of admixed individuals was found in Europe, lower in the Caucasus and Mongolia and the lowest in Yakutia. The variation in introgression rates may be associated with human density and human footprint index (highest in Europe, lowest in Yakutia). Regions with high human densities may be associated with higher number of free‐ranging dogs and thus higher encounter rate between wolves and dogs, which may facilitate admixture. High frequency of wolves carrying signatures of dog admixture in Europe could also result from strong and long‐lasting hunting pressure, which resulted in the local extirpation of wolf populations in large parts of the continent (Dufresnes et al., 2018). Strong hunting pressure is thought to increase the frequency of hybridization due to the disruption to wolf pack structure (Moura et al., 2014; Rutledge et al., 2012) and thus persistent hunting within a given region over many generations may lead to recurrent hybridization and higher admixture proportions.

Hunting and anthropogenic habitat changes have also led to a reduction in wolf population size. In a small wolf population, a single back‐cross event can provide a considerable contribution to the wolf gene pool. Since the size of the domestic dog population is linked to the human population size (Gompper, 2014), the imbalance between wolf and dog population sizes will continue to grow and is likely to lead to further increases in dog introgression into wolves, unless preventive measures are applied (see Donfrancesco et al., 2019; Salvatori et al., 2020).

Based on predictions from ecological studies on the effect of anthropogenic food on large carnivores, attraction to anthropogenic food sources may result in contemporary self‐domestication (Newsome et al., 2017). Hybridization may considerably accelerate this process by enabling rapid acquisition of adaptations facilitating survival in human‐modified landscapes (e.g., increased copy number of *AMY2B* gene facilitating starch digestion— Axelsson et al., 2013). However, areas with frequent human–wolf interactions tend to have a high density of free‐ranging domestic dogs, meaning that the ecological niche of a domesticated canid is already occupied. The increased use of anthropogenic food by wolves may thus result in an increased competition with dogs, which should maintain the niche partitioning between them. Alternatively, wolf and dog populations in such regions may merge into a hybrid swarm, but genetic data from earlier studies consistently show that gene pools of dogs and wolves remain distinct, despite local hybridization (e.g., Hindrikson et al., 2017; Pilot et al., 2018, 2019).

### 4.2 Factors affecting introgression patterns in free‐ranging dogs

In Eurasian FRDs, the wolf admixture proportion was relatively low (0.75%), despite the fact that both FRD males and females are polygamous (Natoli et al., 2021), which should facilitate hybridization and backcrossing. However, the rate of wolf introgression into the FRD gene pool may be limited by the very large dog population sizes relative to wolf population sizes. In a large FRD population, a single back‐cross event, that is successful reproduction of a wolf‐dog hybrid with a dog, will have a limited population‐level effect with regard to the resulting proportion of admixed versus nonadmixed individuals as well as the proportion of hybridization‐derived variants within the entire FRD gene pool. Low wolf admixture proportion in FRDs may also result from the reduced power to detect wolf introgression into dogs compared with dog introgression into wolves. However, in some regions of Eurasia including Saudi Arabia, Mongolia and South‐East Asia, we found high frequencies of dogs carrying a small proportion of introgressed variants (Figure S2). Most of these regions are within wolf's distribution range, with the exception of Thailand. The presence of dogs with wolf admixture in Thailand may result from geographic expansion of introgressed variants beyond the geographic area where hybridization occurs. Alternatively, it could result from cross‐breeding of FRDs with pure‐bred dogs of East Asian origin, which show considerable wolf admixture (e.g., Pilot et al., 2019; Skoglund et al., 2015).

Ancient introgression has been reported both from wolves to dogs (Miao et al., 2017; Skoglund et al., 2015) and in the opposite direction (Bergström et al., 2020), but ancient dog‐to‐wolf introgression has been shown to be considerably more frequent (Bergström et al., 2020). At the early stages of dog domestication, the dog population sizes must have been small, and therefore, backcrossing events, even if rare, must have left a substantial signature in the dog gene pool. This may explain the presence of considerable admixture from a Pleistocene wolf lineage in Arctic and East Asian breeds (Skoglund et al., 2015). In the contemporary FRDs studied here, the average size of introgressed blocks is larger than in wolves, which may suggest that FRDs carry a larger proportion of introgressed blocks originating from recent admixture. However, a number of other factors could have affected the average ancestry block size, including selection on introgressed variants as well as demographic history. Linkage disequilibrium in FRDs is higher than in most wolf populations (e.g., Pilot et al., 2015), and this may affect the size of introgressed blocks.

### 4.3 The effect of recombination on introgression patterns

In many admixed species and subspecies, local admixture proportions are positively correlated with recombination rates (e.g., Janoušek et al., 2015; Martin et al., 2019; Schumer et al., 2018). This is explained by the presence of variants that are deleterious in hybrids, which constitute barriers to introgression, in multiple loci across the genome (Martin et al., 2019). Recombination determines the extent of linkage between a barrier locus and surrounding neutral loci, and loci close to recombination hotspots are expected to have higher admixture proportions than those in regions of low recombination. In the present study, we found no significant correlation between admixture proportions and recombination rates in the genome‐wide analysis and found a significant positive correlation within only a small number of individual chromosomes. We also found few hybrid ancestry deserts (regions with <0.1% of hybrid ancestry), suggesting that the number of “barrier loci” between wolves and FRDs is small. This may be the reason for the observed decoupling of introgression rates from recombination rates. Although wolves and dogs are ecologically distinct, their recent divergence, estimated at about 27–40 thousand years ago (Freedman & Wayne, 2017; Skoglund et al., 2015; Wang, Zhai, et al., 2016), overlapping geographic ranges and continuous admixture, can all contribute to the low occurrence of barriers to introgression.

The observed differences in introgression patterns between wolves and dogs could potentially result from differences in the recombination rates between the two canids. To the best of our knowledge, genome‐wide recombination maps are only available for domestic dogs, not for grey wolves, and we therefore could not test whether differences in recombination rates between wolves and dogs could affect the estimated size of admixed blocks. However, Muñoz‐Fuentes et al. (2015) found no difference in the number and distribution of recombination breakpoints between wolves and dogs within 16 chromosomal regions containing genes associated with phenotypic traits that distinguish dogs from wolves (e.g., coat colour and length, leg length, “dewclaws”). They concluded that recombination patterns were not changed by strong directional selection associated with the domestication process. Therefore, it is unlikely that the shorter average length of admixed blocks in wolves relative to dogs resulted from differences in average recombination rates.

Recombination rate may also affect the selection inference (O'Reilly et al., 2008; Xiang‐Yu et al., 2016). Positive selection was shown to reduce population‐based estimates of recombination rate (O'Reilly et al., 2008) or, conversely, create false recombination hotspots (Reed & Tishkoff, 2006). Moreover, genome‐wide scans to detect signatures of recent selection in humans identified loci located predominantly in regions of low recombination, implying a confounding effect of recombination rate on the power to detect selection (Reed & Tishkoff, 2006). The EHH‐based methods reduce this effect by using genomic distances instead of physical distances, and by contrasting the EHH values of two alleles from each locus, thus removing the effect of local recombination rate (Sabeti et al., 2007; Voight et al., 2006; Xiang‐Yu et al., 2016). Moreover, in our study recombination rates were not correlated with the local admixture proportions and therefore could not bias the inference of positive selection on introgressed variants.

### 4.4 Chromosomal blocks with overrepresented introgressed variants

Although the genome‐wide introgression rate from FRDs into West Eurasian wolves was over three times higher than that from wolves into Eurasian FRDs (2.7% vs. 0.75%), the number and average length of introgressed chromosomal blocks overrepresented in the gene pool were smaller in wolves than FRDs. Of the genes located within these introgressed blocks, the proportion showing signatures of adaptive introgression was four times smaller in wolves than FRDs (5.6% vs. 23%). This implies that introgression resulting from wolf‐dog hybridization yields proportionally larger adaptive advantage to FRDs than wolves. This may be due to the larger population size of FRDs compared to wolves, resulting in the increased efficiency of positive selection on weakly advantageous introgressed variants. An increased level of deleterious variation in dogs compared to wolves (Marsden et al., 2016) may also play a role.

In large FRD populations, genetic drift is weak and therefore gene variants with even a weak selective advantage may increase in frequency. Grey wolf population sizes are considerably smaller and adaptive variants are therefore more likely to be eliminated by drift. However, dogs have gone through multiple bottlenecks during their evolutionary history, including the bottleneck associated with the initial domestication process (Lindblad‐Toh et al., 2005; Ostrander et al., 2017) as well as founder effects associated with expansion events across the world (e.g., Wang, Zhai, et al., 2016). As a result, the genetic variability of FRDs (measured by heterozygosity as well as the number and size of runs of homozygosity) is smaller than that of most wolf populations, although larger compared to that of pure‐bred dogs (Boyko et al., 2010; Marsden et al., 2016). Therefore, FRDs may benefit from introgressive hybridization with wolves by increasing their adaptive variation. Moreover, domestic dogs have a higher genetic load than wolves due to the reduced ability of natural selection to remove weakly deleterious mutations during bottlenecks associated with domestication and breed formation (Marsden et al., 2016). Genetic load is the reduction in average fitness of genotypes in a study population compared to a reference. This reference may be an optimal theoretical genotype, the genotype of an individual with highest fitness in a population or an entire population that has a higher average fitness than the target population. In this case, the wolf population serves as a reference for the dog population. Pure‐bred dogs have on average a two to three per cent greater genetic load than wolves, while the genetic load of FRDs is, as expected, intermediate between wolves and pure‐bred dogs (Marsden et al., 2016). Wolf introgression may therefore result in a reduced genetic load in FRDs, providing an adaptive benefit.

It may be expected that introgression of deleterious variation from FRDs into wolf populations will result in purifying selection, thus creating chromosomal blocks where dog ancestry is underrepresented. We did not, however, detect any blocks with underrepresented dog ancestry in wolves when using a threshold of three SD below the mean, possibly because this threshold was too strict. Using, instead, the criterion of dog ancestry <0.1% (previously used in Sankararaman et al. (2014) to identify Neanderthal ancestry deserts in humans), we identified only two dog ancestry deserts in wolves compared to ten wolf ancestry deserts in FRDs. Although the number of ancestry deserts identified depends on the threshold used to define them, the same threshold applied to wolves and FRDs enables a meaningful comparison of patterns observed in both taxa. Most of the genetic load in dogs is mediated by weakly deleterious mutations that cannot be easily purged from gene pools (Marsden et al., 2016). It is therefore possible that mutations which are weakly deleterious in FRDs remain weakly deleterious when introgressed into wolves, and can thus be maintained in the wolf gene pool instead of being purged, as wolves have low effective population sizes (Fan et al., 2016; Pilot, Greco, et al., 2014; see Table S7) and are thus affected by strong genetic drift.

Recessive mutations of large effect are known to occur in pure‐bred dogs, but they are likely subject to strong purifying selection in FRD populations, and are thus unlikely to be transferred to wolves, as unsupervised hybridization between pure‐bred dogs and wolves is rare. Our result suggests that only a small proportion of variation derived from FRDs has a strongly deleterious effect in wolves. The larger number of wolf ancestry deserts detected in FRDs may results from larger efficiency of selection against deleterious variants in FRDs, due to the larger population size compared to wolves.

Our finding that higher genome‐wide admixture proportions are not necessarily associated with a larger number of blocks with overrepresented hybrid ancestry is consistent with the study by vonHoldt et al. (2016) regarding admixed wolf and coyote populations in North America. They identified 24 blocks with overrepresented coyote ancestry in the Great Lakes wolf population with 14.5% coyote ancestry, but a smaller number (21) of overrepresented blocks in the north‐eastern coyote population with 17.5% wolf ancestry (vonHoldt et al., 2016). This implies that the processes of neutral introgression and adaptive introgression are decoupled, even though both are dependent on demographic patterns in cross‐breeding taxa.

### 4.5 Genes within the chromosomal blocks with overrepresented introgressed variants in wolves

In the West Eurasian wolves, the set of 72 OHA genes (i.e., genes located within the chromosomal blocks characterized by overrepresented dog ancestry) was enriched for the GO terms “amino acid neurotransmitter reuptake” and “glutamate reuptake.” Glutamate is the most abundant excitatory neurotransmitter in the vertebrate central nervous system and accounts for over 90% of synaptic connections in the brain (Platt, 2007). Glutamate receptors play a key role in the induction and maintenance of synaptic plasticity and are associated with learning and memory (Peng et al., 2011). They regulate genomic responses to dopamine stimulation in the neurons of the striatum, a part of the forebrain that coordinates motor functions as well as multiple cognitive functions such as action planning, motivation and reward perception (Wang et al., 2003).

Of 72 OHA genes we identified in West Eurasian wolves, only four (5.6%) showed evidence for adaptive introgression (Table 1), that is were characterized by overrepresentation of dog ancestry and signatures of positive selection. All four genes are involved in neurotransmission and neurodevelopment, and in humans, mutations in these genes are associated with disorders such as schizophrenia, Alzheimer's disease and congenital microcephaly (Table 3). Overall, these results suggest that certain dog‐derived behavioural or cognitive traits may be advantageous to wild wolf populations, highlighting the need for studies on behavioural consequences of wolf‐dog hybridization based on direct observations.

### 4.6 Genes within the chromosomal blocks with overrepresented introgressed variants in free‐ranging dogs

The set of 311 OHA genes from the FRDs included 35 *OR* genes located on three different chromosomes, and 13 protocadherin (*PCDH*) genes clustered on chromosome 2. The presence of multiple genes from the same functional groups is reflected in the results of the GO analysis. OR activity was a significantly enriched molecular function and “homophilic cell adhesion via plasma membrane adhesion molecules,” a type of calcium‐dependent cell adhesion specific to protocadherins, was a significantly enriched biological process.

The efficient function of both *OR* genes and *PCDH* genes is dependent on genetic variation within the gene families (Chen & Maniatis, 2013; Trimmer et al., 2019). Introgression from wolves is likely to increase that variability, thus alleviating the loss of genetic diversity in dogs resulting from the domestication bottleneck. Improvement in FRD olfactory abilities via introgression from wolves may facilitate detection of food sources, identification of unsuitable food, and detection of potential threats (e.g., humans or large predators).

Clustered PCDHs are involved in calcium‐mediated transcriptional gene networks, are expressed primarily in the developing nervous system and play a key role in many neurodevelopmental processes, including axon guidance, creation of new synapses and dendritic self‐avoidance (Garrett & Weiner, 2009; Lefebvre et al., 2012). The organization of the *PCDH* gene cluster enables the expression of multiple gene isoforms, facilitating the diversification of surface molecules in neuronal cells (Chen & Maniatis, 2013). Therefore, clustered PCDHs are considered as “molecular barcodes for self‐recognition by individual neurons in the vertebrate nervous system” (Chen & Maniatis, 2013). It is thus likely that adaptive benefits of wolf introgression in FRDs may result from both introduction of new adaptive variants and an increase in overall diversity of *PCDH* genes. *PCDHs* are under balancing selection in humans (Noonan et al., 2003) and this may be also the case in other vertebrates, which may explain why we found no signatures of positive selection in these genes.

Several studies regarding the genetic basis of animal domestication show that protocadherins displayed differential expression and allele frequency changes between domesticated and wild populations, as well as between populations that were experimentally selected for tame versus aggressive behaviour (Wang et al., 2018). Comparative genomic analyses revealed diversifying selection on several *PCDH* genes between domestic and wild cats (Montague et al., 2014) as well as between tame and aggressive strains of silver foxes (*Vulpes vulpes*) originating from the Farm Fox Experiment (Wang et al., 2018). Genes from *PCDHGA* subfamily displayed differential expression in the brain between strains selected for either tame or aggressive behaviour in both silver foxes and rats (*Rattus norvegicus*) (Heyne et al., 2014; Wang et al., 2018). Tameness in animals is characterized by reduced fear of humans and lowered human‐directed aggression and is, therefore, a necessary trait for wild animals living in close proximity of humans. Although FRDs gain various ecological benefits from living close to human populations, humans are also the main source of early‐life mortality in FRDs (Paul et al., 2016). Extreme tameness associated with a complete lack of fear of humans may therefore lead to reduced fitness. Introgression from wolves could help ensure that tameness in FRD populations does not reach extreme levels where it could become maladaptive.

Interestingly, in wolves we also found overrepresentation of dog variants in a protocadherin gene. This gene, a nonclustered protocadherin *PCDH15*, plays a key role in the formation of sensory hair cells as well as the retina, and variants within this gene are associated with vision and hearing impairment in humans (Jacobson et al., 2008; Kazmierczak et al., 2007). Since we did not identify sequence level variants, we do not know the nature of the resulting functional change in canids. It will be interesting to determine such in subsequent studies, thus providing another example of how studies of canine genome evolution can inform disease studies in humans (see Ostrander, 2012).

Of the 311 OHA genes observed in FRDs, 72 (23%) showed signatures of adaptive introgression (Table 1). The majority of resulting enriched GO terms were associated with calcium channel activity (Table 2). Dysregulation of calcium‐mediated transcriptional gene networks can disrupt the development of neuronal circuits (Kabir et al., 2017; Lohmann, 2009; Ramocki & Zoghbi, 2008). Multiple genes affecting calcium channel activity have been identified in earlier studies as candidate genes for neuropsychiatric disorders in humans and are shown to affect behavioural traits (Table 3 and references therein). Genes affecting behaviour were likely a selection target during the domestication process and introgression of wolf‐derived variants may introduce some wolf‐like behavioural traits, which may be adaptive in free‐living dog populations.

One of the candidate genes under adaptive introgression in FRDs, *TRPC4*, encoding a nonselective calcium‐permeable cation channel, was found to be highly differentiated in Arctic sled dogs relative to other dogs and is therefore hypothesized to play a role in cold‐climate adaptation, together with several other genes involved in calcium ion transport (Sinding et al., 2020). Since our study includes dogs from different climate zones across Eurasia, the adaptive introgression observed on this geographic scale cannot be associated with climate adaptation. This suggests that *TRPC4*, as a gene with pleiotropic effects, could have contributed to both global and local adaptations in dogs.

Among the set of 72 genes showing adaptive introgression signatures, we also found overrepresentation of terms associated with the axoneme, the main structural component of a cilium. Genes associated with axoneme assembly are frequently involved in dyskinesia of primary cilia, which are specialized organelles responsible for calcium signalling within cells that regulate the hedgehog signalling pathways (Delling et al., 2013; Mukhopadhyay & Rohatgi, 2014). Several genes involved in the functioning of primary cilia were previously shown to be under diversifying selection between FRDs and pure‐bred dogs, suggesting their role in the independent survival of free‐living populations (Pilot et al., 2016). This could be related to the role of primary cilia in reproduction (Lee & Gleeson, 2011; Olbrich et al., 2002), and/or their involvement in calcium signalling that affects neurodevelopment. Thus, introgression from wolves may result in the improvement of reproductive fitness of FRDs, or in the introduction of behavioural or morphological traits that may facilitate their independent survival.

The candidate genes discussed above were identified using iHS statistics (Voight et al., 2006) without correction for multiple testing. Two remaining candidate genes under adaptive introgression in FRDs after Bonferroni correction are *HTR2A* and *RYR3*, which are involved in calcium signalling, affect neurobiological processes and are associated with behavioural disorders in humans and other mammals (Matsuo et al., 2009; Serretti et al., 2007; Table 3). Therefore, even after applying a very conservative approach, we can maintain our conclusion that introgression from wolves has a particularly strong effect on behavioural traits in FRDs.

## 5 CONCLUSIONS AND IMPLICATIONS FOR WOLF POPULATION MANAGEMENT

Our results imply that introgressive hybridization with wolves is beneficial for FRD populations, given the evidence of adaptive introgression in multiple genes with important functions. In grey wolves, the introgression is driven mostly by drift, with a small number of positively selected genes being associated with brain function and behaviour. The predominance of drift may be a consequence of the small effective population sizes, resulting in the reduced efficiency of selection on weakly advantageous or against weakly disadvantageous introgressed variants. Variants associated with strong selective disadvantage are likely to be eliminated by natural and sexual selection so that they do not spread from F1 hybrids or recent‐generation backcrosses into further generations, thus preventing the detection of negative selection against them in admixed wolf populations. The scarcity of dog‐derived variants under positive selection implies that wild wolf populations gain little benefits from introgression of domestication‐related traits. However, it can be expected that a larger number of dog‐derived variants could provide an adaptive advantage in wolf populations living in regions highly modified by humans, a topic which warrants further research.

The results of this study have important implications for the management of wolf populations. Given that the neutral introgression rate following a single hybridization event is higher in a small population compared to a large population, and the efficiency of selection for weakly advantageous or against weakly disadvantageous introgressed variants decreases with the decline of population size, the population demography is an important element to consider when planning the strategies to mitigate wolf‐dog hybridization. Management strategies currently being applied or advised to be applied in order to reduce hybridization rate include lethal/nonlethal removal or sterilization of FRDs and hybrids, prevention of poaching and habitat restoration (Donfrancesco et al., 2019; Salvatori et al., 2020). These strategies are aimed at reducing the numbers of admixed individuals present in wolf populations and reducing the frequency of future hybridization events. However, to reduce the introgression rate of dog‐derived variants into the gene pools of wolf populations, it is also essential to maintain as large wolf population sizes as possible.

Finally, our results suggest that genes affecting neurobiological processes predominate among genes displaying higher than average introgression rates in both wolves and FRDs. Therefore, introgressive hybridization can affect behavioural or cognitive traits in both canids. Changes in behaviour of wild wolves can have important ecological consequences, and therefore, monitoring of wolf populations affected by hybridization should include behavioural observations.

## CONFLICT OF INTEREST

The authors declare no conflict of interest.

## Supporting information

Figure S1‐S5

Table S1‐S7

## Data Availability

Data used in this study are available at the following repositories:
Data from Vaysse et al. (2011): http://dogs.genouest.org/SWEEP.dir/Supplemental.html
Data from Cronin et al. (2015): https://doi.org/10.5061/dryad.284tf
Data from Pilot et al. (2015): https://doi.org/10.5061/dryad.078nc
Data from Stronen et al. (2015): https://doi.org/10.5061/dryad.p6598
Data from Frantz et al. (2016): https://doi.org/10.5061/dryad.8gp06
Data from Fitak et al. (2018) (which also includes data from Vernau et al. (2013)): https://doi.org/10.5061/dryad.g68k008
Data from Pilot et al. (2019): https://data.mendeley.com/datasets/4k3yrn3brm/1.
