# Supplementary material for: Human‐modified canids in human‐modified landscapes: The evolutionary consequences of hybridization for grey wolves and free‐ranging domestic dogs
Source: Evol Appl. 2021 Jun 21;14(10):2433–56. doi: 10.1111/eva.13257 (PMC8549620; doi:10.1111/eva.13257)
Supplement: Supplementary file 1 — Figure S1‐S5 [file EVA-14-2433-s001.pdf]

Supplementary Figures

Human-modified canids in human-modified landscapes: the evolutionary consequences of hybridisation for grey wolves and free-ranging domestic dogs

Figure legends

Figure S1. Graphical illustration of the method used to define outlier loci based on within-chromosome standard deviation (SD). X axis shows SNP order along the chromosome, and Y axis shows the admixture proportions. A) Within-chromosome mean lower than global mean (across all loci from 38 autosomal chromosomes). Several loci with highest admixture proportions significantly deviate from the within-chromosome mean (>3SD above the mean). When this 3SD value is applied to the global mean, there are no loci that exceed the threshold value. Therefore, no loci are considered as significant, in order to reduce the number of false positives (since the mean admixture proportion for this chromosome is low, potentially due to the presence of ancestry deserts that reduce the mean). B) Within-chromosome mean higher than global mean. Several loci with highest admixture proportions significantly deviate from the within-chromosome mean (>3SD above the mean). When this 3SD value is applied to the global mean, a larger number of loci exceeds the threshold value. All these loci as considered as significant, in order to reduce the number of false negatives (since the mean admixture proportion for this chromosome is high, potentially due to positive selection on some introgressed variants that increases the mean).

Figure S2. Distribution of admixed and non-admixed Eurasian grey wolves and free-ranging dogs analysed in this study. To provide graphical representation of admixture proportions, we classified individuals as “admixed” if their admixture proportions were above 0.025. Individuals with lower admixture proportions, but carrying introgressed chromosomal blocks, were classified as “individuals with introgressed blocks”. Because some introgressed blocks may be subject to positive selection (as shown in our analyses) and thus spread in a population at a faster rate than neutral blocks, individuals carrying introgressed blocks may be more numerous than expected under a neutral introgression process. Given the low threshold of admixture proportions used to classify individuals classified as “admixed”, they should not be interpreted as “hybrids”. We found only one F1 hybrid (shown on the map). The number of samples belonging to each admixed category collected from the same locations is reflected by the size of the symbol representing this category. Geographic locations of samples are precise except Mongolian wolves and free-ranging dogs from China and Portugal, which have approximate locations. Grey wolf geographic range drawn according to Boitani et al. (2018) and Wang et al. (2016b).

Figure S3. Distribution of dog ancestry in admixed West Eurasian wolves. X axis shows SNP order along each autosomal chromosomes (without reflecting physical distances between SNP loci), and Y axis shows the proportion of dog admixture in wolves (with only admixed individuals considered). The horizontal line represents the mean dog admixture across 38 autosomal chromosomes. Chromosomal blocks with overrepresented dog ancestry are marked in red and are defined as having at least 10 sequential SNPs with the proportion of dog ancestry > 3 SD above the mean, which was calculated across all loci from 38 autosomal chromosomes. Ancestry deserts are marked in orange.

Figure S4. Distribution of wolf ancestry in admixed Eurasian free-ranging dogs (FRDs). X axis shows SNP order along each autosomal chromosomes (without reflecting physical distances between SNP loci), and Y axis shows the proportion of wolf admixture in FRDs (with only admixed individuals considered). The horizontal line represents the mean wolf admixture across 38 autosomal chromosomes. Chromosomal blocks with overrepresented wolf ancestry are marked in red and are defined as having at least 10 sequential SNPs with the proportion of wolf ancestry > 3 SD above the mean, which was calculated across all loci from 38 autosomal chromosomes. Ancestry deserts are marked in orange.

Figure S5. “Randomised chromosomes” constructed to assess the error rate associated with the detection of chromosomal blocks with significant overrepresentation of introgressed alleles in wolves (A) and FRDs (B). Blue colour represents true positive results, red – false positives, orange – false negatives, and black – true negatives.

Figure S1

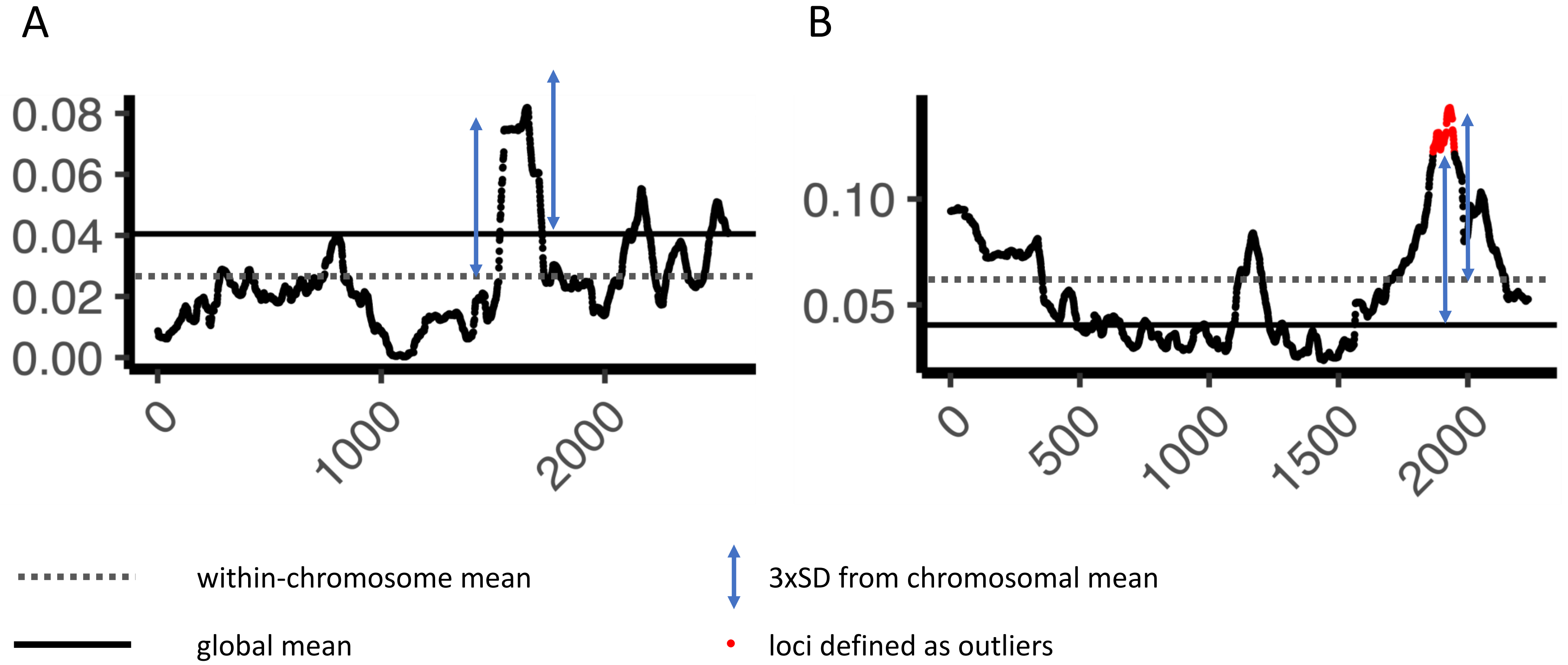

A
B
0.08
0.06
0.04
0.02
0.00
0
1000
2000
0.10
0.05
0
500
1000
1500
2000
within-chromosome mean
global mean
3xSD from chromosomal mean
loci defined as outliers

Figure S2

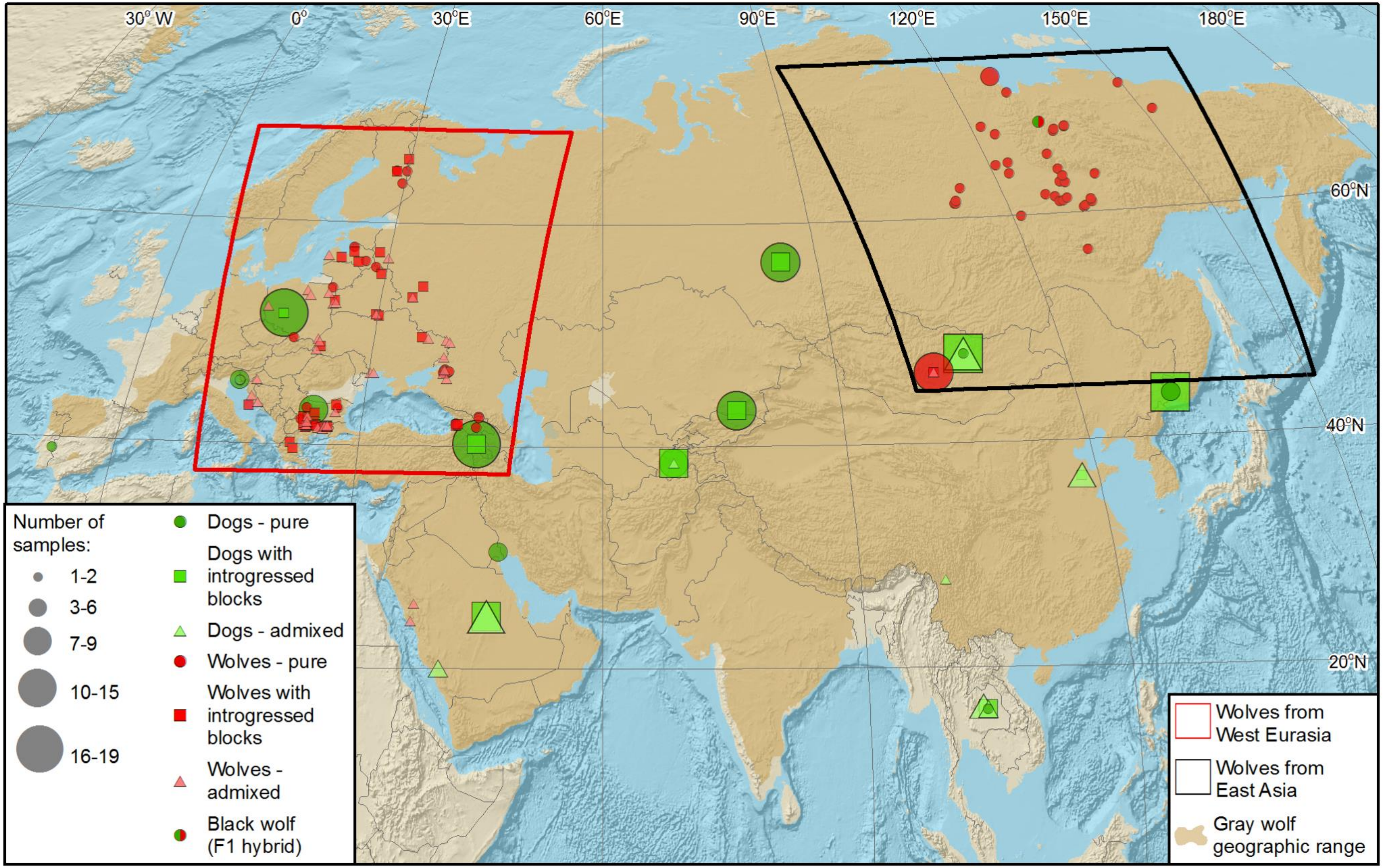
30° W
0°
30°E
60°E
90°E
120°E
150°E
180°E
60°N
40°N
20°N
Number of samples:
1-2
3-6
7-9
10-15
16-19
Dogs - pure
Dogs with introgressed blocks
Dogs - admixed
Wolves - pure
Wolves with introgressed blocks
Wolves - admixed
Black wolf (F1 hybrid)
Wolves from West Eurasia
Wolves from East Asia
Gray wolf geographic range

Figure S3

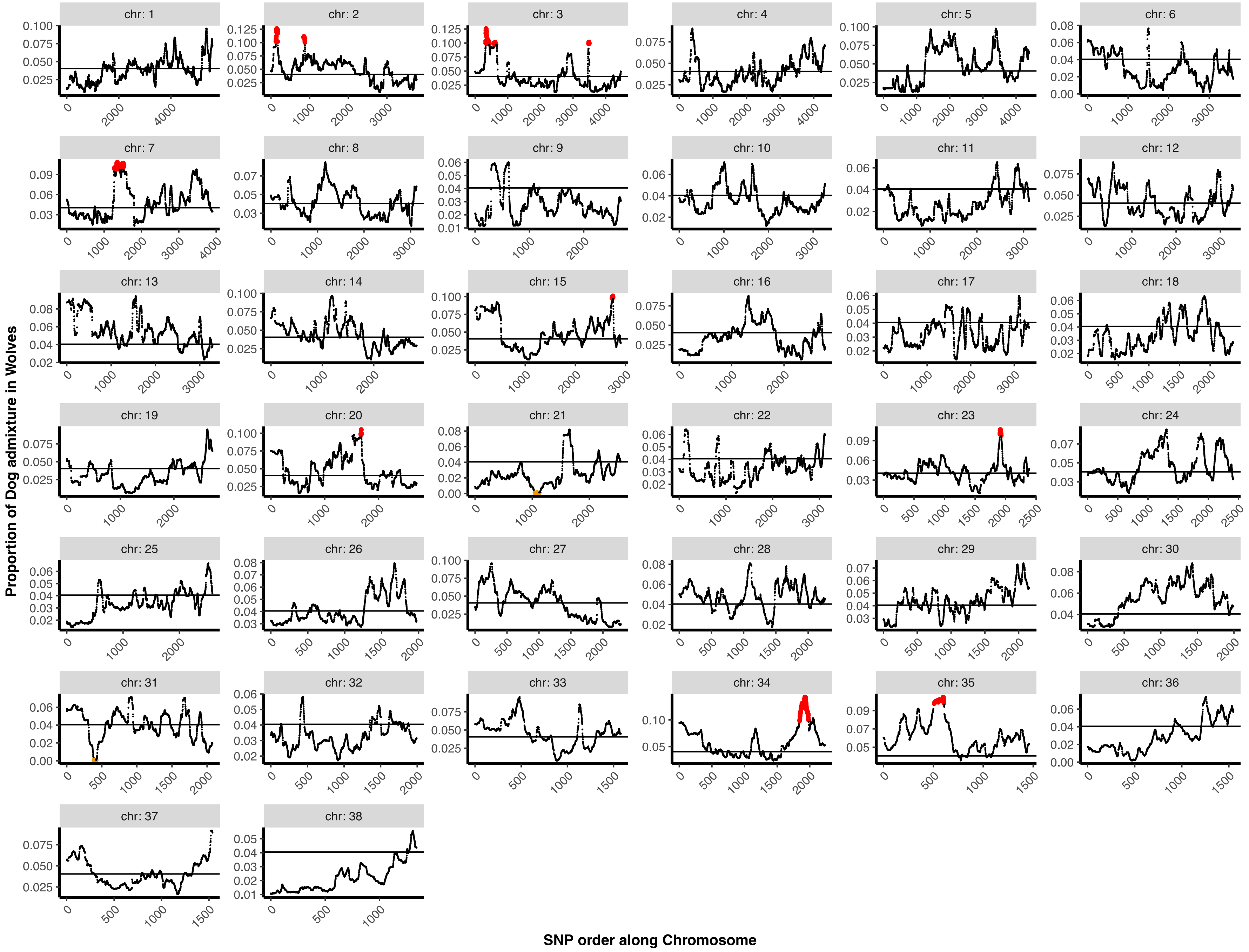
chr: 1
chr: 2
chr: 3
chr: 4
chr: 5
chr: 6
chr: 7
chr: 8
chr: 9
chr: 10
chr: 11
chr: 12
chr: 13
chr: 14
chr: 15
chr: 16
chr: 17
chr: 18
chr: 19
chr: 20
chr: 21
chr: 22
chr: 23
chr: 24
chr: 25
chr: 26
chr: 27
chr: 28
chr: 29
chr: 30
chr: 31
chr: 32
chr: 33
chr: 34
chr: 35
chr: 36
chr: 37
chr: 38
Proportion of Dog admixture in Wolves
SNP order along Chromosome

Figure S4

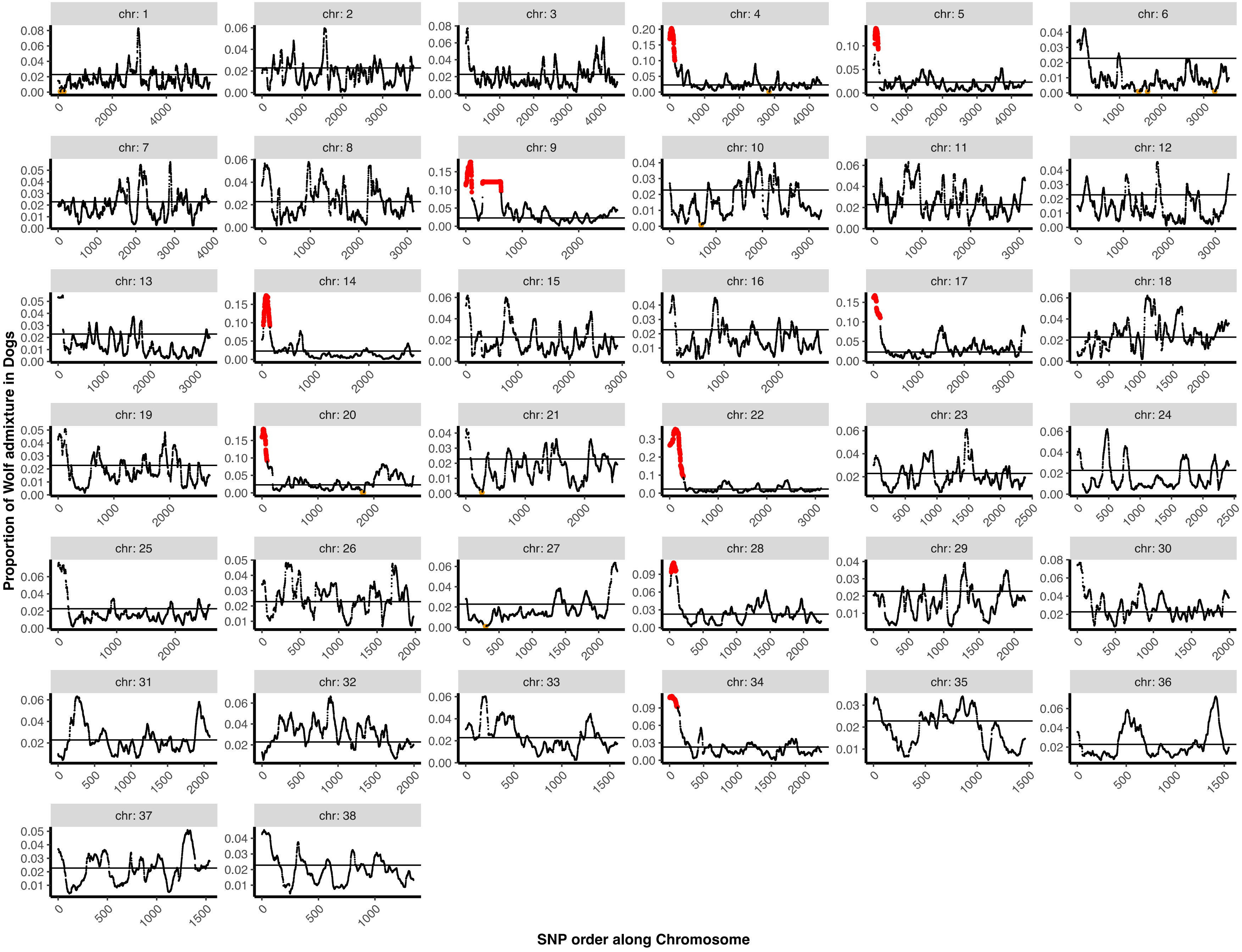

chr: 1
chr: 2
chr: 3
chr: 4
chr: 5
chr: 6
chr: 7
chr: 8
chr: 9
chr: 10
chr: 11
chr: 12
chr: 13
chr: 14
chr: 15
chr: 16
chr: 17
chr: 18
chr: 19
chr: 20
chr: 21
chr: 22
chr: 23
chr: 24
chr: 25
chr: 26
chr: 27
chr: 28
chr: 29
chr: 30
chr: 31
chr: 32
chr: 33
chr: 34
chr: 35
chr: 36
chr: 37
chr: 38
Proportion of Wolf admixture in Dogs
SNP order along Chromosome

Figure S5

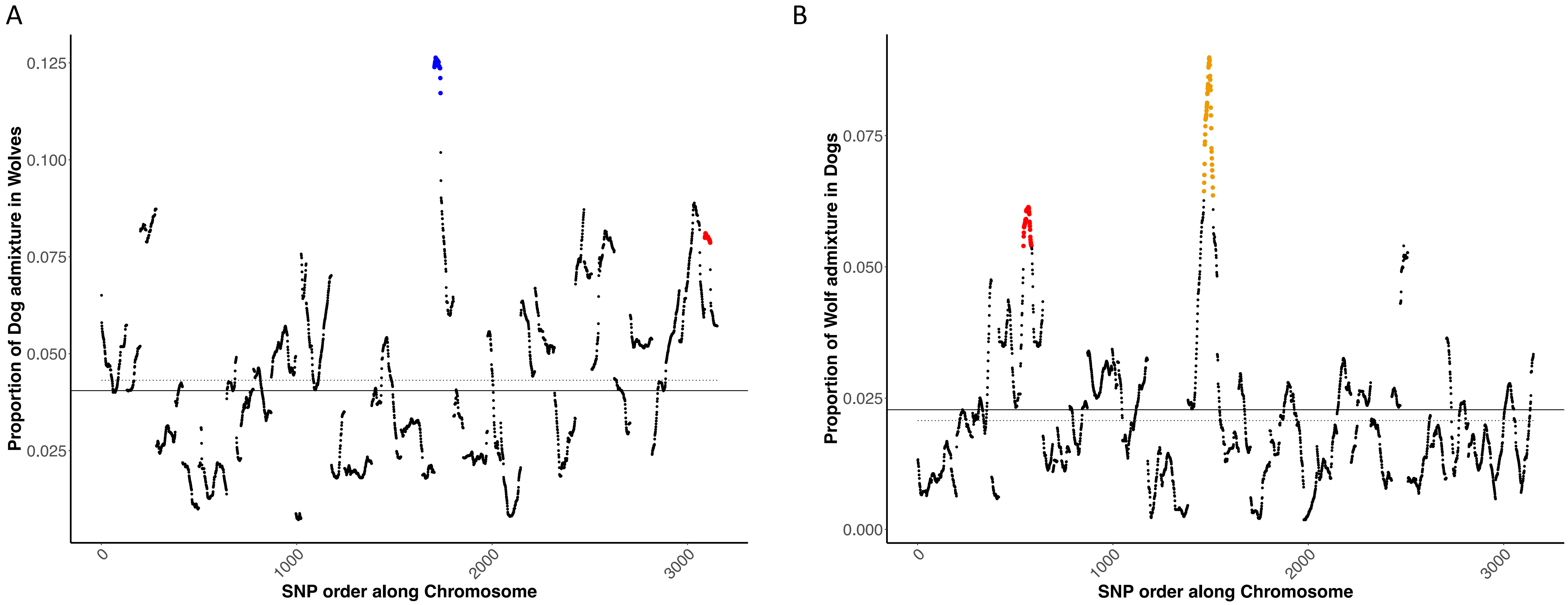
A
Proportion of Dog admixture in Wolves
0.125
0.100
0.075
0.050
0.025
0
1000
2000
3000
SNP order along Chromosome
B
Proportion of Wolf admixture in Dogs
0.075
0.050
0.025
0.000
0
1000
2000
3000
SNP order along Chromosome
