# Supplementary material for: Human‐modified canids in human‐modified landscapes: The evolutionary consequences of hybridization for grey wolves and free‐ranging domestic dogs
Source: Evol Appl. 2021 Jun 21;14(10):2433–56. doi: 10.1111/eva.13257 (PMC8549620; doi:10.1111/eva.13257)
Supplement: Supplementary file 2 — Table S1‐S7 [file EVA-14-2433-s002.pdf]

SUPPLEMENTARY TABLES

Human-modified canids in human-modified landscapes: the evolutionary consequences of hybridisation for grey wolves and free-ranging domestic dogs

Małgorzata Pilot, Andre E. Moura, Innokentiy M. Okhlopkov, Nikolay V. Mamaev, Ninna H. Manaseryan, Vahram Hayrapetyan, Natia Kopaliani, Elena Tsingarska, Abdulaziz N. Alagaili, Osama B. Mohammed, Elaine A. Ostrander, Wiesław Bogdanowicz

Table S1. List of samples included in the admixture analyses. The last column indicates samples that were subsequently used in the analyses of adaptive introgression (AI).

Canid	Country	N	Source	Used in the AI analysis
wolves	Georgia	11	Pilot et al. 2019	yes
wolves	Mongolia	14	Pilot et al. 2019	
wolves	Nagorny Karabakh	15	Pilot et al. 2019	yes
wolves	Saudi Arabia	2	Pilot et al. 2019	
wolves	Yakutia, Russia	41	Pilot et al. 2019	
wolves	Bulgaria	38	Pilot et al. 2019	yes
wolves	Belarus	8	Stronen et al. 2013	yes
wolves	Bulgaria	2	Stronen et al. 2013	yes
wolves	Croatia	4	Stronen et al. 2013	yes
wolves	Finland	5	Stronen et al. 2013	yes
wolves	Greece	2	Stronen et al. 2013	yes
wolves	Latvia	6	Stronen et al. 2013	yes
wolves	Poland	8	Stronen et al. 2013	yes
wolves	Russia	5	Stronen et al. 2013	yes
wolves	Slovakia	1	Stronen et al. 2013	yes
wolves	Ukraine	16	Stronen et al. 2013	yes
wolves	British Columbia	35	Cronin et al. 2015	
wolves	Idaho	23	Cronin et al. 2015	
wolves	Interior Alaska	40	Cronin et al. 2015	
wolves	Minnesota	20	Cronin et al. 2015	
wolves	Montana	16	Cronin et al. 2015	
wolves	New Mexico	8	Cronin et al. 2015	
wolves	Southeast Alaska	138	Cronin et al. 2015	
wolves	Wyoming	25	Cronin et al. 2015	
wolves	New Mexico, Arizona	87	Fitak et al. 2018	
wolves	Europe	12	Vaysse et al. 2011	yes
wolves	North America	1	Vaysse et al. 2011	
free-ranging dogs	Armenia	25	Pilot et al. 2015	yes
free-ranging dogs	Bulgaria	9	Pilot et al. 2015	yes
free-ranging dogs	Central Russia	16	Pilot et al. 2015	yes
free-ranging dogs	China	9	Pilot et al. 2015	yes
free-ranging dogs	East Russia	19	Pilot et al. 2015	yes
free-ranging dogs	Iraq	8	Pilot et al. 2015	yes

free-ranging dogs	Kazakhstan	20	Pilot et al. 2015	yes
free-ranging dogs	Mongolia	27	Pilot et al. 2015	yes
free-ranging dogs	Poland	21	Pilot et al. 2015	yes
free-ranging dogs	Saudi Arabia	27	Pilot et al. 2015	yes
free-ranging dogs	Slovenia	13	Pilot et al. 2015	yes
free-ranging dogs	Tajikistan	19	Pilot et al. 2015	yes
free-ranging dogs	Thailand	21	Pilot et al. 2015	yes
free-ranging dogs	China	6	Frantz et al. 2015	yes
free-ranging dogs	India	2	Frantz et al. 2015	yes
free-ranging dogs	Portugal	2	Frantz et al. 2015	yes
pure-bred dogs (various breeds)	N/A	446	Vaysse et al. 2011	
pure-bred dogs (various breeds)	N/A	192	Pilot et al. 2015	
pure-bred dogs (2 breeds)	Asia	12	Frantz et al. 2015	
mixed-breed dogs	North America	36	Fitak et al. 2018	
mixed-breed dogs (Alaskan husky)	Alaska	10	Vernau et al. 2013	

Table S2. Correlations between local admixture proportions (DAP – in dogs; WAP – in wolves) and recombination rate (RR), and between the admixture proportions at the same loci in dogs versus wolves. The table presents Pearson's correlation coefficients, with significant values (assessed using the linear regression model) marked in bold, and stars representing the significance levels: *<0.05; **<0.01; ***<0.001. Negative correlations are highlighted in grey.

chromosome	DAP vs RR	WAP s RR	DAP vs WAP
1	-0.044	0.099	**-0.098***
2	0.014	**-0.118***	-0.015
3	-0.022	-0.025	**-0.264*****
4	0.059	**0.100***	**0.191*****
5	0.018	-0.002	**0.128****
6	-0.039	0.001	0.046
7	-0.053	-0.042	-0.056
8	-0.025	0.030	**0.167****
9	-0.054	-0.024	**0.243*****
10	0.066	**0.145****	-0.033
11	-0.072	**0.108***	**-0.191*****
12	-0.041	0.014	-0.094
13	0.026	-0.012	0.115
14	0.046	-0.106	-0.011
15	-0.049	-0.012	**-0.252*****
16	0.022	-0.075	-0.035
17	-0.106	-0.082	**0.313*****
18	0.019	0.007	0.013
19	0.044	**0.121***	**-0.167****
20	-0.050	0.017	**-0.222*****
21	0.014	0.030	**0.303*****
22	0.006	**0.178****	-0.041
23	-0.060	-0.026	**-0.181****
24	0.033	0.048	**-0.150***
25	-0.041	0.073	**0.212*****
26	**-0.123***	0.099	**0.122***
27	0.023	0.013	**-0.568*****
28	-0.024	0.050	**-0.332*****
29	-0.065	0.038	-0.009
30	0.053	-0.064	-0.064
31	0.095	-0.190	**-0.362*****
32	**0.153***	**-0.154***	**-0.224*****
33	-0.056	0.009	**0.278*****
34	-0.026	-0.002	**0.266*****
35	-0.062	-0.024	0.001
36	0.089	0.109	0.235
37	0.123	0.031	**0.260*****
38	0.078	-0.002	**-0.424*****
genome-wide	-0.004	0.010	**-0.042*****

Table S3. Chromosomal blocks with overrepresentation of introgressed ancestry in wolves and FRDs, identified based on the global standard deviation from the mean calculated across all loci from 38 autosomal chromosomes (as opposed to Table 1, where local outliers were identified based on standard deviation within each chromosome).

Population	chr	Chromosomal block position	Block size	N SNP loci
wolves	2	3508922-7373539	3864617	48
wolves	2	21701739-22575749	874010	35
wolves	3	9035020-11291899	2256879	110
wolves	3	14501405-15333660	832255	54
wolves	3	74837475-75458693	621218	20
wolves	7	27355517-27952557	597040	20
wolves	7	28880506-29515468	634962	46
wolves	7	29863095-30308120	445025	33
wolves	7	31449399-32519364	1069965	51
wolves	15	60808514-61242530	434016	22
wolves	20	34956448-35106018	149570	12
wolves	23	43743063-44113271	370208	22
wolves	34	35326046-37636703	2310657	137
wolves	35	8895111-10453398	1558287	97
wolves	mean		1144194	50.5
FRDs	4	43598-3844859	3801261	149
FRDs	5	1559873-3379769	1819896	97
FRDs	9	13741-3011941	2998200	106
FRDs	9	9003519-16745118	7741599	329
FRDs	14	1960408-4898061	2937653	121
FRDs	17	51048-3686421	3635373	150
FRDs	20	144948-3726204	3581256	94
FRDs	22	108262-6525885	6417623	282
FRDs	28	571534-2166613	1595079	74
FRDs	34	127067-2342162	2215095	115
FRDs	mean		3674304	151.7

Table S4. Chromosomal blocks with underrepresentation of introgressed ancestry in wolves and FRDs. The criteria used to determine underrepresentation were the frequency of introgressed variants <0.1% and at least 10 SNPs included within the block.

Population	chr	Chromosomal block position	Block size	Average introgressed ancestry	N genes	N SNP loci
wolves	21	21-21030297 – 21-23033131	2002834	0.00055	20/20	72
wolves	31	31-7369975 – 31-7849487	479512	0.00052	1/2	30
FRDs	1	1-2948208 – 1-3697160	748952	0.00074	3/3	10
FRDs	1	1-6520082 – 1-6865364	345282	0.00052	0/0	22
FRDs	4	4-59919172 – 4-60081615	162443	0.00073	1/1	15
FRDs	6	6-32813125 – 6-33307813	494688	0.00088	2/2	26
FRDs	6	6-38053050 – 6-38853551	800501	0.00080	36/42	19
FRDs	6	6-70963470 – 6-71298555	335085	0.00080	1/1	16
FRDs	10	10-18936594 – 10-19166470	229876	0.00072	1/1	19
FRDs	20	20-37800623 – 20-38699338	898715	0.00047	10/12	29
FRDs	21	21-7585240 – 21-7929971	344731	0.00058	2/3	15
FRDs	27	27-5091565 – 27-5432794	341229	0.00090	7/8	16

N genes – number of genes within the chromosomal block; two values reported represent N genes annotated in the canine genome / N orthologous genes annotated in the human genome identified in the synteny analysis
N SNPs – number of genotyped SNPs within the chromosomal block

Table S5. Results of Gene Ontology analysis carried out for two sets of genes in wolves and free-ranging dogs. The analysis was carried out for canine genes using the dog reference genome and for the human orthologues using the human reference genome. The significance were determined using the Benjamini-Hochberg correction (BH) and the more conservative g:SCS (Set Counts and Sizes) false-discovery rate correction method. Only P-values below 0.05 are shown. OEA – the set of genes located within chromosomal blocks with overrepresented exogenous ancestry; AI – the set of genes showing signatures of adaptive introgression.

Gene set	Canid	Reference genome	GO source	Term name	Term ID	P (BH)	P (g:SCS)	Term size	Query size	Intersection size
OEA	dog	human	GO:MF	RNA-DNA hybrid ribonuclease activity	GO:0004523	0.0399		8	278	3
OEA	dog	human	GO:MF	histidine-tRNA ligase activity	GO:0004821	0.0399		2	278	2
OEA	dog	human	GO:MF	olfactory receptor activity	GO:0004984	0.0399		396	278	17
OEA	dog	human	GO:MF	endoribonuclease activity, producing 5'-phosphomonoesters	GO:0016891	0.0399	0.0417	31	278	5
OEA	dog	human	GO:MF	single-stranded RNA binding	GO:0003727	0.0482		86	278	7
OEA	dog	human	GO:BP	homophilic cell adhesion via plasma membrane adhesion molecules	GO:0007156	0.0062	0.0033	167	271	13
OEA	dog	human	GO:BP	cell-cell adhesion via plasma-membrane adhesion molecules	GO:0098742	0.0414	0.0443	276	271	15
OEA	dog	dog	GO:MF	calcium ion binding	GO:0005509	0.0226		549	203	20
OEA	dog	dog	GO:MF	cation binding	GO:0043169	0.0226		1693	203	43
OEA	dog	dog	GO:MF	metal ion binding	GO:0046872	0.0226		1637	203	42
OEA	dog	dog	GO:MF	histidine-tRNA ligase activity	GO:0004821	0.0254		2	203	2
OEA	dog	dog	GO:MF	ion binding	GO:0043167	0.0299		3395	203	71
OEA	dog	dog	GO:BP	homophilic cell adhesion via plasma membrane adhesion molecules	GO:0007156	0.0000	0.0000	81	195	13
OEA	dog	dg	GO:BP	cell-cell adhesion via plasma-membrane adhesion molecules	GO:0098742	0.0000	0.0000	121	195	14
OEA	dog	dog	GO:BP	cell-cell adhesion	GO:0098609	0.0052	0.0317	384	195	19
OEA	dog	dog	GO:BP	cell adhesion	GO:0007155	0.0228		641	195	24
OEA	dog	dog	GO:BP	biological adhesion	GO:0022610	0.0228		646	195	24
OEA	dog	dog	GO:BP	ribonucleoside metabolic process	GO:0009119	0.0390		31	195	5
OEA	wolf	human	GO:MF	ATP-dependent peptidase activity	GO:0004176	0.0305	0.0497	5	59	2
OEA	wolf	dog	GO:BP	amino acid neurotransmitter reuptake	GO:0051933	0.0145	0.0368	3	40	2

OEA	wolf	dog	GO:BP	glutamate reuptake	GO:0051935	0.0145	0.0368	3	40	2
AI	dog	human	GO:MF	ryanodine-sensitive calcium-release channel activity	GO:0005219	0.0139	0.0472	4	67	2
AI	dog	human	GO:MF	calcium-induced calcium release activity	GO:0048763	0.0139	0.0472	4	67	2
AI	dog	human	GO:BP	sequestering of calcium ion	GO:0051208	0.0232	0.0285	142	66	6
AI	dog	human	GO:BP	maintenance of location in cell	GO:0051651	0.0232	0.0486	233	66	7
AI	dog	human	GO:CC	calcium channel complex	GO:0034704	0.0297	0.0255	67	67	4
AI	dog	human	GO:CC	junctional sarcoplasmic reticulum membrane	GO:0014701	0.0463		9	67	2
AI	dog	human	GO:CC	sarcoplasmic reticulum membrane	GO:0033017	0.0463		40	67	3
AI	dog	dog	GO:MF	ryanodine-sensitive calcium-release channel activity	GO:0005219	0.0090	0.0140	3	51	2
AI	dog	dog	GO:MF	calcium channel activity	GO:0005262	0.0109	0.0338	66	51	4
AI	dog	dog	GO:MF	calcium ion transmembrane transporter activity	GO:0015085	0.0178		83	51	4
AI	dog	dog	GO:MF	metal ion transmembrane transporter activity	GO:0046873	0.0342		301	51	6
AI	dog	dog	GO:MF	calcium-release channel activity	GO:0015278	0.0342		11	51	2
AI	dog	dog	GO:MF	ligand-gated calcium channel activity	GO:0099604	0.0493		16	51	2
AI	dog	dog	GO:MF	protein serine/threonine kinase inhibitor activity	GO:0030291	0.0493		18	51	2
AI	dog	dog	GO:MF	intracellular ligand-gated ion channel activity	GO:0005217	0.0493		17	51	2
AI	dog	dog	GO:MF	ARF guanyl-nucleotide exchange factor activity	GO:0005086	0.0493		16	51	2
AI	dog	dog	GO:BP	axoneme assembly	GO:0035082	0.0157	0.0191	38	51	4
AI	dog	dog	GO:BP	axonemal dynein complex assembly	GO:0070286	0.0245		18	51	3
AI	dog	dog	GO:BP	microtubule bundle formation	GO:0001578	0.0247		56	51	4
AI	dog	dog	GO:BP	regulation of ventricular cardiac muscle cell action potential	GO:0098911	0.0329		8	51	2
AI	dog	dog	GO:BP	calcium ion homeostasis	GO:0055074	0.0329		168	51	5
AI	dog	dog	GO:BP	atrial cardiac muscle cell action potential	GO:0086014	0.0329		7	51	2
AI	dog	dog	GO:BP	AV node cell action potential	GO:0086016	0.0329		7	51	2
AI	dog	dog	GO:BP	cell projection assembly	GO:0030031	0.0329		255	51	6

AI	dog	dog	GO:BP	cellular cation homeostasis	GO:0030003	0.0329		251	51	6
AI	dog	dog	GO:BP	cellular metal ion homeostasis	GO:0006875	0.0329		236	51	6
AI	dog	dog	GO:BP	cellular ion homeostasis	GO:0006873	0.0329		258	51	6
AI	dog	dog	GO:BP	calcium ion transmembrane transport	GO:0070588	0.0329		133	51	5
AI	dog	dog	GO:BP	atrial cardiac muscle cell to AV node cell signaling	GO:0086026	0.0329		7	51	2
AI	dog	dog	GO:BP	AV node cell to bundle of His cell signaling	GO:0086027	0.0329		7	51	2
AI	dog	dog	GO:BP	atrial cardiac muscle cell to AV node cell communication	GO:0086066	0.0329		7	51	2
AI	dog	dog	GO:BP	AV node cell to bundle of His cell communication	GO:0086067	0.0329		8	51	2
AI	dog	dog	GO:BP	cellular calcium ion homeostasis	GO:0006874	0.0329		164	51	5
AI	dog	dog	GO:BP	metal ion homeostasis	GO:0055065	0.0344		263	51	6
AI	dog	dog	GO:BP	cellular divalent inorganic cation homeostasis	GO:0072503	0.0380		178	51	5
AI	dog	dog	GO:BP	cell-cell signaling involved in cardiac conduction	GO:0086019	0.0418		12	51	2
AI	dog	dog	GO:BP	divalent inorganic cation homeostasis	GO:0072507	0.0418		186	51	5
AI	dog	dog	GO:BP	outer dynein arm assembly	GO:0036158	0.0418		11	51	2
AI	dog	dog	GO:BP	sequestering of calcium ion	GO:0051208	0.0418		48	51	3
AI	dog	dog	GO:BP	regulation of cardiac muscle cell action potential	GO:0098901	0.0418		12	51	2
AI	dog	dog	GO:BP	calcium ion transport	GO:0006816	0.0418		194	51	5
AI	dog	dog	GO:BP	cation homeostasis	GO:0055080	0.0418		293	51	6
AI	dog	dog	GO:BP	inorganic ion homeostasis	GO:0098771	0.0447		299	51	6
AI	dog	dog	GO:BP	ventricular cardiac muscle cell action potential	GO:0086005	0.0499		14	51	2
AI	dog	dog	GO:BP	epithelial cilium movement involved in extracellular fluid movement	GO:0003351	0.0499		14	51	2
AI	wolf	human	GO:CC	cell junction	GO:0030054	0.0144	0.0360	2099	4	4
AI	wolf	human	GO:CC	lamellipodium	GO:0030027	0.0314		201	4	2
AI	wolf	dog	none	-	-	-		-	-	-

Table S6. SNP loci located within chromosomal blocks with significant overrepresentation of introgressed ancestry in free-ranging dogs (FRDS) and wolves, which show significant results in the iHS test for positive selection at P<0.05. Only SNPs located within protein-coding genes are listed. The table lists the position of each SNP on a chromosome, a gene within which the SNP is located, the value of the iHS statistic, the corresponding P-value and the threshold P-value after Bonferroni correction, which accounts for all SNPs within a chromosome used in the test. Only two SNPs have significant P-values after the Bonferroni correction.

Population	Chr	SNP position	Gene	iHS	P-value	P-value after Bonferroni correction	significant after correction
FRDs	1	61770970	SERINC1	2.6499	0.0080508	9.218E-06	
FRDs	1	61787479	SERINC1/HSF2	2.9399	0.0032829	9.218E-06	
FRDs	1	61807469	SERINC1/HSF2	2.6005	0.0093094	9.218E-06	
FRDs	1	62059918	PKIB	-2.5180	0.0118010	9.218E-06	
FRDs	1	62094560	SMPDLA3	-2.5217	0.0116789	9.218E-06	
FRDs	1	62255573	CL2S2	-2.3921	0.0167534	9.218E-06	
FRDs	1	62584220	TRDN	-2.0898	0.0366313	9.218E-06	
FRDs	2	35816009	IK cytokine	2.0639	0.0390293	1.369E-05	
FRDs	2	35843728	HARS2	2.1289	0.0332632	1.369E-05	
FRDs	2	35860575	HARS2	2.6286	0.0085733	1.369E-05	
FRDs	2	36073089	PCDHA13	-2.4744	0.0133455	1.369E-05	
FRDs	3	660857	EPB41L4A	2.1525	0.0313562	1.154E-05	
FRDs	4	2377011	RYR2	-2.6010	0.0092946	1.194E-05	
FRDs	4	2442049	RYR2	-2.4556	0.0140668	1.194E-05	
FRDs	4	2457135	RYR2	-2.5203	0.0117253	1.194E-05	
FRDs	4	2533448	RYR2	-2.3907	0.0168155	1.194E-05	
FRDs	4	2559001	RYR2	-2.8692	0.0041157	1.194E-05	
FRDs	4	2600929	RYR2	-2.3680	0.0178837	1.194E-05	
FRDs	4	2857922	RYR2	-2.0882	0.0367815	1.194E-05	
FRDs	4	3293805	MTR	-2.3996	0.0164121	1.194E-05	
FRDs	5	2291313	OPCML	2.0940	0.0362594	1.175E-05	
FRDs	5	2321049	OPCML	2.1084	0.0349995	1.175E-05	
FRDs	5	2810094	NTM	2.8474	0.0044077	1.175E-05	
FRDs	5	3322000	NTM	2.0736	0.0381119	1.175E-05	
FRDs	9	850630	SLC38A10	3.2129	0.0013141	1.938E-05	
FRDs	9	916372	AATK	2.6179	0.0088472	1.938E-05	
FRDs	9	935245	BAIAP2	3.5502	0.0003850	1.938E-05	
FRDs	9	977060	BAIAP2	3.3896	0.0006999	1.938E-05	
FRDs	9	980264	BAIAP2	3.3896	0.0006999	1.938E-05	
FRDs	9	1139989	RPTOR	2.7447	0.0060575	1.938E-05	
FRDs	9	1176860	RPTOR	2.7155	0.0066182	1.938E-05	
FRDs	9	1188547	RPTOR	2.6990	0.0069557	1.938E-05	
FRDs	9	1259288	RPTOR	2.3564	0.0184521	1.938E-05	
FRDs	9	1300807	RPTOR	2.3521	0.0186689	1.938E-05	

FRDs	9	1454911	RNF213	2.7288	0.0063574	1.938E-05
FRDs	9	1455021	RNF213	2.7288	0.0063574	1.938E-05
FRDs	9	1507833	RNF213	-2.3832	0.0171649	1.938E-05
FRDs	9	1533048	SLC26A11	2.6774	0.0074203	1.938E-05
FRDs	9	1614108	GAA	2.6549	0.0079338	1.938E-05
FRDs	9	1669133	TBC1D16	-2.8781	0.0040003	1.938E-05
FRDs	9	1725998	TBC1D16	-2.7458	0.0060371	1.938E-05
FRDs	9	2605238	CYTH1	-2.2765	0.0228170	1.938E-05
FRDs	9	2632358	CYTH1	-2.1829	0.0290446	1.938E-05
FRDs	9	2719467	DNAH17	-2.0708	0.0383732	1.938E-05
FRDs	14	3001892	AKR1B1	-2.7437	0.0060745	1.820E-05
FRDs	14	3232879	LRGUK	2.0880	0.0367954	1.820E-05
FRDs	14	3249578	LRGUK	2.9653	0.0030242	1.820E-05
FRDs	14	3264856	LRGUK	2.2440	0.0248298	1.820E-05
FRDs	14	3864949	EXOC4	2.7448	0.0060557	1.820E-05
FRDs	14	4339415	CHCHD3	2.1598	0.0307851	1.820E-05
FRDs	14	4432850	CHCHD3	2.8054	0.0050261	1.820E-05
FRDs	14	4478077	CHCHD3	2.2251	0.0260770	1.820E-05
FRDs	14	4569454	CHCHD3	2.4288	0.0151493	1.820E-05
FRDs	14	4609934	CHCHD3	2.7120	0.0066884	1.820E-05
FRDs	17	92369	FAM110C	2.6826	0.0073042	1.544E-05
FRDs	17	206370	SH3YL1	-2.5014	0.0123695	1.544E-05
FRDs	17	214944	SH3YL1	-2.0866	0.0369250	1.544E-05
FRDs	17	272432	ENSCAFG00000042100	-2.2892	0.0220661	1.544E-05
FRDs	17	1883263	EIPR1	2.0560	0.0397786	1.544E-05
FRDs	17	1911686	EIPR1	2.0797	0.0375564	1.544E-05
FRDs	17	1958951	EIPR1	2.2847	0.0223303	1.544E-05
FRDs	17	2120962	RNASEH1	2.3855	0.0170573	1.544E-05
FRDs	17	2143263	RPS7	2.3983	0.0164708	1.544E-05
FRDs	17	2328291	DCDC2C	2.5100	0.0120739	1.544E-05
FRDs	20	1786438	MGLL	2.7532	0.0059018	1.901E-05
FRDs	20	3115640	ACAD9	2.5266	0.0115187	1.901E-05
FRDs	20	3406396	IQSEC1	2.6931	0.0070800	1.901E-05
FRDs	22	191461	ATP7B	-2.7679	0.0056416	1.647E-05
FRDs	22	222969	ATP7B	-3.0080	0.0026294	1.647E-05
FRDs	22	310310	WDFY2	2.4440	0.0145243	1.647E-05
FRDs	22	316765	WDFY2	2.9587	0.0030897	1.647E-05
FRDs	22	426979	WDFY2	-2.1408	0.0322887	1.647E-05
FRDs	22	575684	INTS6	2.1196	0.0340357	1.647E-05
FRDs	22	642583	SERPINE3	2.2746	0.0229309	1.647E-05
FRDs	22	799416	ENSCAFG00000004287	2.3662	0.0179698	1.647E-05
FRDs	22	1889131	SNPYD7	-2.1056	0.0352377	1.647E-05
FRDs	22	1917806	SNPYD7	-2.4190	0.0155615	1.647E-05
FRDs	22	1938990	SNPYD7	-2.2616	0.0237239	1.647E-05

FRDs	22	3222187	RB1	-2.0548	0.0398983	1.647E-05	
FRDs	22	3245315	RB1	2.8254	0.0047227	1.647E-05	
FRDs	22	4513532	HTR2A	4.6015	0.0000042	1.647E-05	yes
FRDs	22	4567366	ESD	2.3661	0.0179789	1.647E-05	
FRDs	25	2214097	FREM2	3.4543	0.0005518	2.028E-05	
FRDs	25	2393702	UFM1	-2.0932	0.0363323	2.028E-05	
FRDs	25	2456285	UFM1	-2.7475	0.0060043	2.028E-05	
FRDs	25	2495696	UFM1	-2.0539	0.0399838	2.028E-05	
FRDs	25	2507108	UFM1	-2.3512	0.0187138	2.028E-05	
FRDs	25	2533627	UFM1	-2.3915	0.0167782	2.028E-05	
FRDs	25	3084236	TRPC4	-2.7801	0.0054338	2.028E-05	
FRDs	25	3131334	TRPC4	-3.3017	0.0009612	2.028E-05	
FRDs	25	3184738	POSTN	-3.0482	0.0023020	2.028E-05	
FRDs	25	3660731	SUPT20H	-2.5709	0.0101442	2.028E-05	
FRDs	25	3791879	SMAD9	-2.3303	0.0197912	2.028E-05	
FRDs	25	4300875	SPART	2.7718	0.0055745	2.028E-05	
FRDs	25	4367633	DCLK1	-2.6644	0.0077128	2.028E-05	
FRDs	25	4379806	DCLK1	-2.3900	0.0168481	2.028E-05	
FRDs	25	4387638	DCLK1	-2.3900	0.0168481	2.028E-05	
FRDs	25	4430801	DCLK1	-3.3440	0.0008259	2.028E-05	
FRDs	25	4457751	DCLK1	-3.6440	0.0002685	2.028E-05	
FRDs	25	4482831	DCLK1	-2.5905	0.0095839	2.028E-05	
FRDs	25	4523030	DCLK1	-3.8921	0.0000994	2.028E-05	
FRDs	25	4531327	DCLK1	-3.4500	0.0005605	2.028E-05	
FRDs	25	4562697	DCLK1	-2.2793	0.0226510	2.028E-05	
FRDs	25	4576087	DCLK1	-4.0281	0.0000562	2.028E-05	
FRDs	25	4614777	DCLK1	3.6889	0.0002252	2.028E-05	
FRDs	25	4630619	DCLK1	-2.9832	0.0028529	2.028E-05	
FRDs	25	4644607	DCLK1	-3.5671	0.0003609	2.028E-05	
FRDs	27	44242348	CACNA1C	2.9696	0.0029820	2.267E-05	
FRDs	27	44254253	CACNA1C	-2.0261	0.0427585	2.267E-05	
FRDs	27	44277996	CACNA1C	3.7718	0.0001621	2.267E-05	
FRDs	27	44292368	CACNA1C	-2.5404	0.0110721	2.267E-05	
FRDs	27	44344170	CACNA1C	2.3397	0.0192975	2.267E-05	
FRDs	27	44367154	CACNA1C	2.0751	0.0379767	2.267E-05	
FRDs	27	44412333	CACNA1C	2.0938	0.0362755	2.267E-05	
FRDs	27	44576806	CACNA1C	2.2818	0.0225005	2.267E-05	
FRDs	27	44626361	CACNA1C	2.0841	0.0371543	2.267E-05	
FRDs	27	44662360	CACNA1C	2.3672	0.0179247	2.267E-05	
FRDs	27	44666232	CACNA1C	2.2436	0.0248564	2.267E-05	
FRDs	27	44685367	CACNA1C	2.7466	0.0060223	2.267E-05	
FRDs	27	44693656	CACNA1C	2.5946	0.0094709	2.267E-05	
FRDs	27	44740972	CACNA1C	2.1619	0.0306264	2.267E-05	
FRDs	28	488365	ARHGAP22	2.5112	0.0120311	2.256E-05	

FRDs	28	846479	WDFY4	-2.4945	0.0126118	2.256E-05	
FRDs	28	859388	WDFY4	-2.3273	0.0199485	2.256E-05	
FRDs	28	862469	WDFY4	-2.2774	0.0227603	2.256E-05	
FRDs	28	895806	WDFY4	-2.2353	0.0253975	2.256E-05	
FRDs	28	965210	VSTM4	-2.5951	0.0094572	2.256E-05	
FRDs	28	1008600	VSTM4	2.3682	0.0178739	2.256E-05	
FRDs	28	1040331	VSTM4	-2.0876	0.0368342	2.256E-05	
FRDs	28	1067568	VSTM4	-2.5542	0.0106444	2.256E-05	
FRDs	28	1758363	PARG	2.7724	0.0055640	2.256E-05	
FRDs	28	1783113	ENSCAFG00000029477	2.8194	0.0048116	2.256E-05	
FRDs	28	1824452	NCOA4	2.3547	0.0185398	2.256E-05	
FRDs	30	833206	SLC12A6	-2.0599	0.0394115	1.701E-05	
FRDs	30	911676	KATNBL1	-2.2300	0.0257453	1.701E-05	
FRDs	30	1176413	AVEN	-3.0314	0.0024343	1.701E-05	
FRDs	30	1189366	RYR3	-3.5328	0.0004112	1.701E-05	
FRDs	30	1363393	RYR3	-3.4503	0.0005599	1.701E-05	
FRDs	30	1376764	RYR3	-3.8168	0.0001352	1.701E-05	
FRDs	30	1411781	RYR3	-3.3800	0.0007249	1.701E-05	
FRDs	30	1532825	RYR3	-4.9226	0.0000009	1.701E-05	yes
FRDs	30	1819062	FMN1	-3.2325	0.0012271	1.701E-05	
FRDs	32	17135664	SMARCAD1	-2.0581	0.0395850	2.589E-05	
FRDs	34	275438	TRIO	2.5386	0.0111296	2.288E-05	
FRDs	34	362889	TRIO	2.3084	0.0209761	2.288E-05	
FRDs	34	390463	TRIO	2.5936	0.0094978	2.288E-05	
FRDs	34	504743	TRIO	4.1251	0.0000371	2.288E-05	
FRDs	34	816274	DNAH5	-2.4909	0.0127414	2.288E-05	
FRDs	34	887911	DNAH5	-2.1939	0.0282452	2.288E-05	
FRDs	34	892511	DNAH5	-3.2658	0.0010915	2.288E-05	
FRDs	34	938929	DNAH5	-2.4071	0.0160789	2.288E-05	
FRDs	34	1002199	DNAH5	-2.2702	0.0231925	2.288E-05	
FRDs	34	1009623	DNAH5	-3.4757	0.0005096	2.288E-05	
wolves	2	22262126	FRMD4A	2.6883	0.0071825	1.681E-05	
wolves	2	6985594	ABI1	3.0582	0.0022264	1.681E-05	
wolves	2	7205932	APBB1IP	2.4700	0.0135104	1.681E-05	
wolves	2	7234111	APBB1IP	2.3506	0.0187424	1.681E-05	
wolves	34	35914984	PLD1 (6Kb upstream)	-2.1282	0.0333242	2.784E-05	

Table S7. Estimates of effective population sizes of regional wolf and dog populations in Eurasia from published studies, obtained using the G-PhoCS method (Gronau et al. 2011) or an LD-based method (Tenesa et al. 2007).

Population	Effective population size	Data type	Method	Source
Tibetan wolf	2,500	whole genome sequence	G-PhoCS	Fan et al. 2016
Inner Mongolian wolf	9,400	whole genome sequence	G-PhoCS	Fan et al. 2016
Xinjiang Wolf	20,800	whole genome sequence	G-PhoCS	Fan et al. 2016
Qinghai wolf	93,700	whole genome sequence	G-PhoCS	Fan et al. 2016
Russian wolf	13,500	whole genome sequence	G-PhoCS	Fan et al. 2016
Croatian wolf	4,600	whole genome sequence	G-PhoCS	Fan et al. 2016
Israeli wolf	16,600	whole genome sequence	G-PhoCS	Fan et al. 2016
Indian wolf-Iranian wolf	6,200	whole genome sequence	G-PhoCS	Fan et al. 2016
Chinese indigenous dog	26,100	whole genome sequence	G-PhoCS	Fan et al. 2016
Eastern European wolves	1,366	SNP chip (61 K SNPs)	LD	Pilot et al. 2014
West Eurasian FRDs	2,538	SNP chip (147 K SNPs)	LD	Pilot et al. 2015

References

Fan, Z., Silva, P., Gronau, I., Wang, S., Armero, A. S., Schweizer, R. M., et al. (2016). Worldwide patterns of genomic variation and admixture in gray wolves. *Genome Research*, 26(2), 163-173.

Gronau I, Hubisz MJ, Gulko B, Danko CG, Siepel A. (2011). Bayesian inference of ancient human demography from individual genome sequences. *Nature Genetics*, 43, 1031–1034.

Pilot, M., Greco, C., Jędrzejewska, B., Randi, E., Jędrzejewski, W., Sidorovich, V. E., et al. (2014b). Genome-wide signatures of population bottlenecks and diversifying selection in European wolves. *Heredity*, 112(4), 428-442.

Pilot, M., Malewski, T., Moura, A. E., Grzybowski, T., Oleński, K., Ruść, A., et al. (2015). On the origin of mongrels: evolutionary history of free-breeding dogs in Eurasia. *Proceedings of the Royal Society B: Biological Sciences*, 282(1820), 20152189.

Tenesa A, Navarro P, Hayes BJ, Duffy DL, Clarke GM, Goddard ME et al. (2007). Recent human effective population size estimated from linkage disequilibrium. *Genome Research*, 17, 520–526.
